# ICP0 Dismantles Microtubule Networks in Herpes Simplex Virus-Infected Cells

**DOI:** 10.1371/journal.pone.0010975

**Published:** 2010-06-08

**Authors:** Mingyu Liu, Edward E. Schmidt, William P. Halford

**Affiliations:** 1 Department of Microbiology and Immunology, Southern Illinois University School of Medicine, Springfield, Illinois, United States of America; 2 Department of Veterinary Molecular Biology, Montana State University, Bozeman, Montana, United States of America; University of California San Francisco, United States of America

## Abstract

Infected-cell protein 0 (ICP0) is a RING finger E3 ligase that regulates herpes simplex virus (HSV) mRNA synthesis, and strongly influences the balance between latency and replication of HSV. For 25 years, the nuclear functions of ICP0 have been the subject of intense scrutiny. To obtain new clues about ICP0's mechanism of action, we constructed HSV-1 viruses that expressed GFP-tagged ICP0. To our surprise, both GFP-tagged and wild-type ICP0 were predominantly observed in the cytoplasm of HSV-infected cells. Although ICP0 is exclusively nuclear during the immediate-early phase of HSV infection, further analysis revealed that ICP0 translocated to the cytoplasm during the early phase where it triggered a previously unrecognized process; ICP0 dismantled the microtubule network of the host cell. A RING finger mutant of ICP0 efficiently bundled microtubules, but failed to disperse microtubule bundles. Synthesis of ICP0 proved to be necessary and sufficient to disrupt microtubule networks in HSV-infected and transfected cells. Plant and animal viruses encode many proteins that reorganize microtubules. However, this is the first report of a viral E3 ligase that regulates microtubule stability. Intriguingly, several cellular E3 ligases orchestrate microtubule disassembly and reassembly during mitosis. Our results suggest that ICP0 serves a dual role in the HSV life cycle, acting first as a nuclear regulator of viral mRNA synthesis and acting later, in the cytoplasm, to dismantle the host cell's microtubule network in preparation for virion synthesis and/or egress.

## Introduction

Herpes simplex virus 1 (HSV-1) is a large, dsDNA virus that is capable of alternating between two programs of gene expression that lead to a productive or silent infection. One of HSV's immediate-early (IE) proteins, ICP0, is a positive regulator whose synthesis represents a key step by which HSV “decides” whether or not an infection is likely to culminate in the production of new infectious virus (reviewed in Ref. [1]). For example, ICP0 is sufficient to trigger HSV reactivation in latently infected trigeminal ganglion neurons [2], [3]. At multiplicities of infection (MOI) above 1 pfu per cell, HSV *ICP0*-deficient (*ICP0* ^−^) viruses replicate to nearly wild-type levels. In contrast, at MOIs below 0.1 pfu per cell, the same *ICP0* ^−^ viruses establish quiescent infections in 99% of the cells they infect [4], [5], [6].

ICP0's mechanism of action is unknown, but the possibilities are constrained by four facts: ***1.*** ICP0 potentiates ICP4's function as a transcriptional activator of HSV mRNA synthesis [7], [8], [9]; ***2.*** ICP0 is a RING-finger E3 ubiquitin ligase [10], [11]; ***3.*** ICP0 is essential for HSV's resistance to the innate interferon response of animals [1], [12]; and ***4.*** ICP0's E3 ubiquitin ligase activity triggers the dispersal of pro-myelocytic leukemia (PML) nuclear bodies [13], [14], which may contribute to the formation of adjacent, sub-nuclear replication compartments [15], [16].

Point mutations in ICP0's RING-finger domain (amino acids 116 to 156) destroy ICP0's E3 ligase activity and destroy ICP0's capacity to promote HSV replication [11]. It remains unclear which substrate(s) explain how ICP0's E3 ligase activity promotes HSV replication and spread. Although ICP0 triggers the efficient dispersal of PML nuclear bodies, ICP0 does not ubiquitinate the PML protein in an *in vitro* E3 ligase assay [17].

Like many laboratories, we have been interested in learning how ICP0 influences outcomes of HSV infection. Our most recent study clarifies that ICP0 physically interacts with HSV's major transcriptional regulator, ICP4, and suggests that ICP0 influences whether ICP4 functions predominantly as an activator or a repressor of HSV mRNA synthesis [9]. However, ICP0's interaction with ICP4 does not explain ***1.*** how ICP0 triggers the dispersal of PML nuclear bodies and centromere proteins [14], [18], nor does it explain ***2.*** why synthesis of ICP0 causes cells to arrest in the G2/M phase of the cell cycle [19], [20]. These latter observations suggest that ICP0 must interact with at least one cellular protein. Rather than interrogate specific proteins for their capacity to interact with ICP0, we chose to use live-cell imaging to determine if new clues might be obtained by tracking the distribution of a green fluorescent protein (GFP)-tagged form of ICP0 in HSV-infected cells over time.

Three *ICP0^+^* viruses were constructed that bore an ∼750 bp insertion of *GFP* coding sequence inserted in the *ICP0* gene. The resulting recombinant viruses, HSV-1 0^+^GFP_12_, HSV-1 0^+^GFP_24_, and HSV-1 0^+^GFP_105_ each synthesized a 3.5 kb ICP0^+GFP^ mRNA and a 140 kDa protein. Each GFP-tagged ICP0 protein retained much of ICP0's activity, and was visible in HSV-1 infected cells by fluorescent microscopy. Contrary to our initial expectations, the majority of GFP-tagged ICP0 was observed in the cytoplasm of HSV-1 infected cells. Subsequent tests verified that wild-type ICP0 also accumulated to much higher levels in the cytoplasm than in the nucleus of virus-infected cells.

Since the discovery that ICP0 potently stimulates HSV mRNA synthesis [7], [8], sporadic reports have documented the presence of ICP0 in the cytoplasm of HSV-infected cells, or have described ICP0 translocating from the nucleus to the cytoplasm of HSV-infected cells [21], [22], [23], [24], [25]. In recent years, ICP0's presence in the cytoplasm of HSV-infected cells has become undeniable, and it is now well established that ICP0 is incorporated into the tegument of HSV-1 virions [26], [27], [28], [29]. However, it remains unclear if ICP0 mediates a specific function in the cytoplasm of HSV-1 infected cells, or if ICP0 translocates to the cytoplasm for the sole purpose of its incorporation into HSV-1 virions [26], [27], [28], [29]. Therefore, most studies of ICP0 continue to focus on deciphering how this E3 ligase functions in the nucleus to stimulate HSV mRNA synthesis [9], [30], [31], [32], [33], [34].

The results of the current study clarify that ICP0 is an exclusively nuclear protein during the IE phase of HSV infection, but translocates to the cytoplasm during the early (E) phase. Once in the cytoplasm of HSV-infected cells, ICP0 efficiently bundles and disperses host cell microtubules. Microtubule networks are known to be disrupted in HSV-infected cells [35], but the effectors that mediate this process are unknown. The HSV-1 tegument protein VP22 bundles microtubules when overexpressed in transfected cells [36], but this is not observed in HSV-infected cells [37]. Our results demonstrate that ICP0 is necessary and sufficient to trigger the complete disassembly of the host cell microtubule network. This finding adds to a growing list of proteins encoded by plant and animal viruses that reorganize microtubules [38], [39], [40], [41].

This is the first report of a viral E3 ligase that regulates microtubule stability. Intriguingly, cellular E3 ligases such as the anaphase-promoting complex and cullin 3 orchestrate massive reorganizations of microtubules during mitosis [42], [43], [44]. ICP0-like E3 ligases are not unique to herpes simplex virus, but are encoded by at least 20 other α-herpesviruses that infect primates, pigs, dogs, kangaroos, and other species [45], [46]. The results of the current study suggest that these α-herpesvirus-encoded E3 ligases fulfill a dual function of ***1.*** immediately stimulating viral gene expression in the nucleus, and ***2.*** acting later, in the cytoplasm, to dismantle the host cell's microtubule network in preparation for virion synthesis and/or egress.

## Results

### Characterization of HSV viruses that express GFP-tagged ICP0

**Figure 1:**
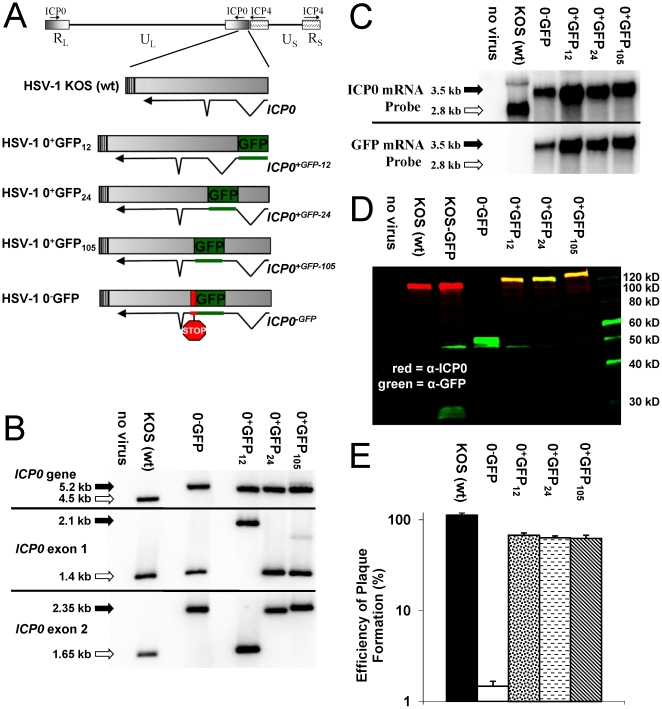
Characterization of HSV-1 viruses that express GFP-tagged ICP0. (A) Schematic of the wild-type or chimeric *ICP0* genes in wild-type HSV-1 strain KOS or the recombinant viruses, HSV-1 0^+^GFP_12_, 0^+^GFP_24_, and 0^+^GFP_105_, and 0^−^GFP. (B) Southern blot analysis of the *ICP0* locus of HSV-1 KOS versus HSV-1 recombinant viruses based on hybridization of an *ICP0* intron1-specific probe to DNA digested with StuI and HpaI (*ICP0* gene), StuI and BamHI (exon 1 fragment), or PshAI and BssHII (exon 2 fragment). (C) Northern blot analysis with an *ICP0*- versus *GFP*-specific probes hybridized to 10 µg total RNA isolated from Vero cells that were uninfected (no virus) or were infected with the specified HSV-1 viruses (MOI = 10; RNA harvested 12 hours p.i.). (D) Two-color Western blot analysis of cells that were uninfected (no virus) or were infected with the specified HSV-1 viruses (MOI = 10; protein harvested 18 hours p.i.). (E) Efficiency of plaque formation by HSV-1 KOS, 0^-^GFP, 0^+^GFP_12_, 0^+^GFP_24_, or 0^+^GFP_105_ in Vero cells relative to the number of plaques that formed in ICP0-complementing L7 cells (mean ± sem; n = 3 cultures per group).

Three *ICP0^+GFP^* genes were constructed that encoded GFP-tagged ICP0 in which ***i.*** GFP replaced amino acids 1 to 11 of the 775-amino acid ICP0 protein (ICP0^+GFP-12^); ***ii.*** GFP was inserted between amino acids 23 and 24 of ICP0 (ICP0^+GFP-24^); or ***iii.*** GFP was inserted between amino acids 104 and 105 of ICP0 (ICP0^+GFP-105^) (Fig. 1A). An ICP0-null control gene, the *ICP0^−GFP^* gene, encoded only the N-terminal 104 amino acids of ICP0 fused to GFP (Fig. 1A). Chimeric *ICP0^GFP^* genes were introduced into the *LAT-ICP0* locus of HSV-1 strain KOS by homologous recombination to yield HSV-1 0^−^GFP, 0^+^GFP_12_, 0^+^GFP_24_, or 0^+^GFP_105_ (Fig. 1B). Northern blot analysis verified that each of these viruses synthesized the predicted 3.5 kb ICP0^GFP^ mRNA (Fig. 1C). GFP-tagged ICP0 proteins were analyzed by two-color Western blot analysis. In cells infected with wild-type HSV-1 KOS or KOS-GFP, ICP0-specific monoclonal antibody H1083 labeled the 110 kDa ICP0 protein (red band in Fig. 1D). In addition, the GFP-expressing recombinant virus, KOS-GFP [47], also encoded an ∼30 kDa GFP protein that was labeled by rabbit anti-GFP antibody (green band in Fig. 1D). HSV-1 0^−^GFP encoded a truncated ∼55 kDa protein that was only labeled by the GFP-specific antibody, whereas the 0^+^GFP viruses encoded ∼140 kDa ICP0^+GFP-12, -24, and -105^ proteins that were labeled with both ICP0- and GFP-specific antibodies (yellow bands in Fig. 1D).

The functionality of ICP0^+GFP-12, -24, and -105^ proteins was compared to ICP0. Wild-type HSV-1 formed plaques with 100% efficiency in Vero cells relative to the number of plaques that formed in ICP0-complementing L7 cells (Fig. 1E). In contrast, only ∼1% of HSV-1 0^−^GFP (*ICP0* ^−^) viruses formed plaques in Vero cells (Fig. 1E). Each HSV-1 0^+^GFP virus formed plaques in Vero cells at ∼66% efficiency relative to the number of plaques that formed in ICP0-complementing L7 cells (Fig. 1E). Thus, ICP0^+GFP-12, -24, or -105^ protein each retained sufficient activity to promote HSV-1 plaque formation with 45-fold greater efficiency than the *ICP0* ^−^ null control virus, HSV-1 0^−^GFP.

### GFP-tagged ICP0 is predominantly observed in the cytoplasm of HSV-infected cells

**Figure 2:**
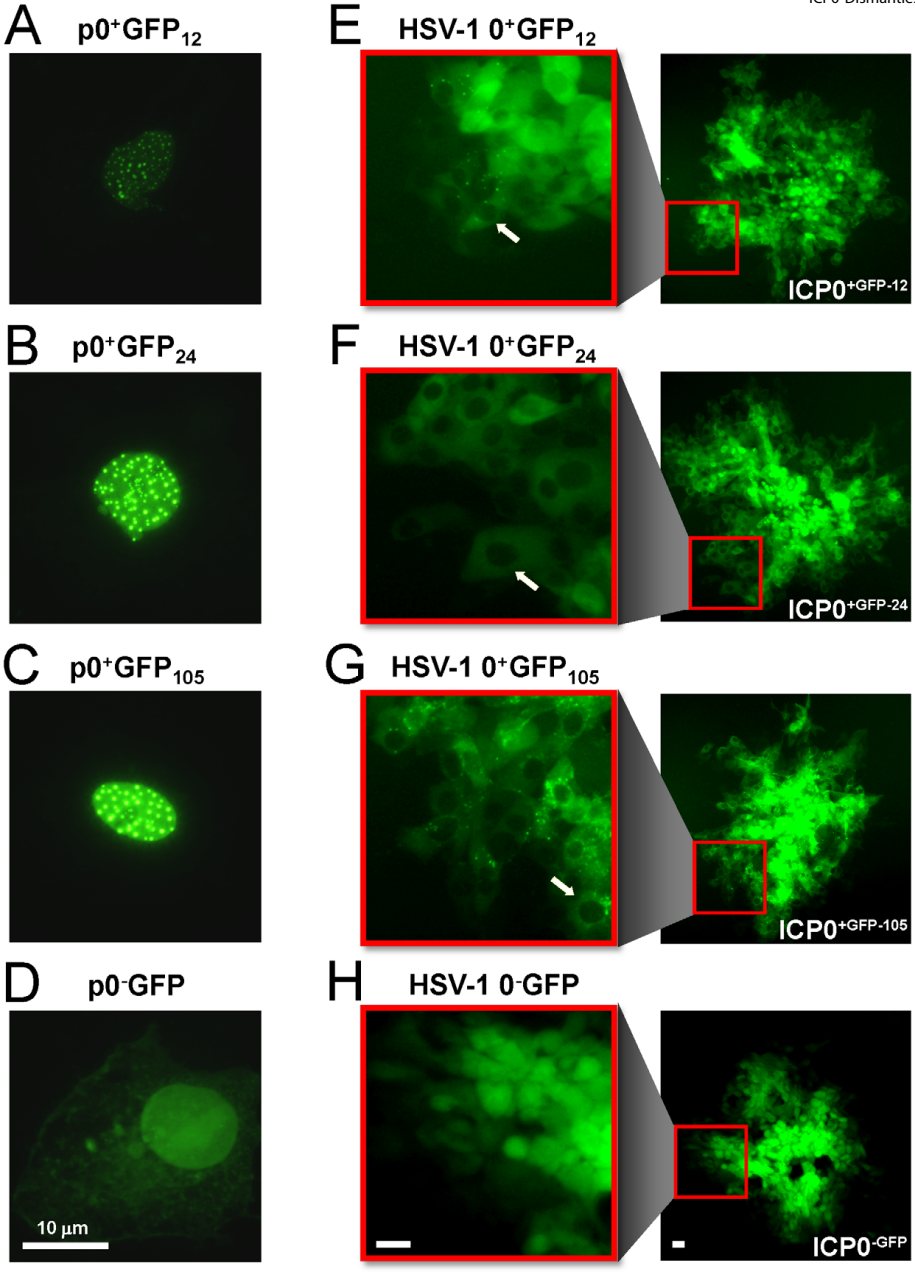
GFP-tagged ICP0 is predominantly cytoplasmic in HSV-1 infected cells. (A) ICP0^+GFP-12^, (B) ICP0^+GFP-24^, (C) ICP0^+GFP-105^, or (D) ICP0^−GFP^ as seen in Vero cells 12 hours post transfection with the plasmids p0^+^GFP_12_, p0^+^GFP_24_, p0^+^GFP_105_, or p0^−^GFP, respectively. Each plasmid was co-transfected with the plasmid pBHad.CMV-VP16 to induce the *ICP0* promoter and expression of GFP-tagged ICP0. (E) ICP0^+GFP-12^, (F) ICP0^+GFP-24^, (G) ICP0^+GFP-105^, or (H) ICP0^−GFP^ as seen in Vero cells 40 hours post inoculation with the HSV-1 recombinant viruses 0^+^GFP_12_, 0^+^GFP_24_, 0^+^GFP_105_, or 0^−^GFP, respectively. In each panel, an entire plaque is shown on the right, and one edge of the plaque is magnified on the left. White arrows denote HSV-1 infected cells in which GFP-tagged ICP0 was abundant in the cytoplasm, but was not evident in the nuclei of HSV-1 infected cells. The scale bar denotes a distance of 10 µm.

In cells transfected with the plasmids p0^+^GFP_12_, p0^+^GFP_24_, or p0^+^GFP_105_, the ICP0^+GFP-12, -24, or -105^ proteins each accumulated in the nuclei of transfected cells in a punctate pattern, typical of wild-type ICP0 (Fig. 2A–C; [13], [14]). In contrast, the truncated ICP0^−GFP^ protein was distributed in a nuclear and perinuclear pattern in cells transfected with the plasmid p0^−^GFP (Fig. 2D). When expressed in the context of HSV-1 infection, ICP0^+GFP-12, -24, or -105^ protein was predominantly observed in the cytoplasm of virus-infected cells. For example, ICP0^+GFP-12^ was predominantly observed in the cytoplasm of cells at the outer edge of plaques formed by HSV-1 0^+^GFP_12_ (white arrow in Fig. 2E). Likewise, the ICP0^+GFP-24^ and ICP0^+GFP-105^ proteins were primarily detected in the cytoplasm of cells at the outer edge of plaques formed by HSV-1 0^+^GFP_24_ and HSV-1 0^+^GFP_105_, respectively (white arrows in Fig. 2F, 2G). In contrast, the ICP0^−GFP^ peptide expressed by HSV-1 0^−^GFP accumulated throughout virus-infected cells (Fig. 2H).

**Figure 3:**
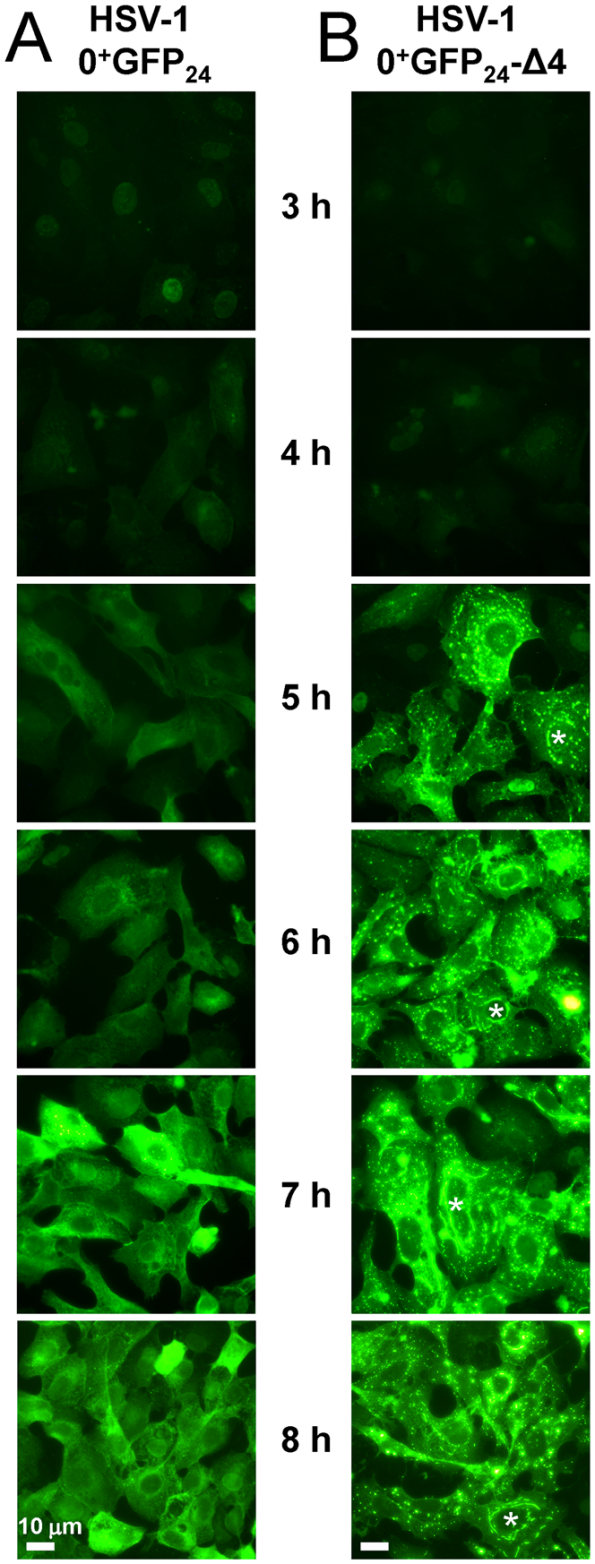
ICP0^+GFP-24^ translocates to the cytoplasm of HSV-1 infected cells in the presence or absence of ICP4. ICP0^+GFP-24^ protein in Vero cells inoculated with 10 pfu per cell of HSV-1 0^+^GFP_24_ (left column) or HSV-1 0^+^GFP_24_-Δ4 (right column) between 3 and 8 hours p.i. White asterisks denote cells in which ICP0^+GFP-24^ was observed in a linear pattern encircling the nucleus of HSV-1 infected cells. The scale bar denotes a distance of 10 µm.

The subcellular distribution of ICP0^+GFP-24^ protein was monitored in Vero cells inoculated with 10 pfu per cell of HSV-1 0^+^GFP_24_. At 3 hours p.i., GFP-tagged ICP0 was evident in the nuclei of many cells infected with HSV-1 0^+^GFP_24_ (Fig. 3A). However, ICP0^+GFP-24^ only accumulated to high levels after 5 hours p.i., at which time the protein had translocated to the cytoplasm of most HSV-1 0^+^GFP_24_-infected cells (Fig. 3A). To determine if the translocation of ICP0^+GFP-24^ was replication-dependent, an HSV-1 *ICP4* ^−^ null virus was constructed, HSV-1 0^+^GFP_24_-Δ4. In cells inoculated with HSV-1 0^+^GFP_24_-Δ4, the nuclear-to-cytoplasmic translocation of ICP0^+GFP-24^ occurred with kinetics similar to the *ICP4* ^+^ virus (Fig. 3B). ICP0^+GFP-24^ was observed in bright perinuclear lines and globular bodies when overexpressed from the *ICP4* ^−^ null virus (Fig. 3B). Intriguingly, this pattern of cytoplasmic ICP0 was equivalent to the findings of Knipe and Smith (1986) [21], who first described ICP0's cytoplasmic distribution in cells inoculated with an HSV-1 *ICP4* mutant. Live-cell imaging of HSV-1 0^+^GFP_24_-Δ4-infected cells demonstrated that the cytoplasmic bodies in which ICP0^+GFP-24^ protein accumulated were highly mobile (Video S1). In addition, cytoplasmic ICP0^+GFP-24^ was often observed in linear arrays that encircled the nucleus (denoted by asterisks in Fig. 3B).

These observations raised questions both about the requirements for ICP0's nuclear-to-cytoplasmic translocation, and what function ICP0 might fulfill in the cytoplasm of HSV-1 infected cells. Further experiments were performed to address these questions.

### Cytoplasmic translocation of ICP0 is an early event in the HSV-1 replication cycle

Although HSV-1 *ICP4* ^−^ null viruses overexpress viral IE proteins, a considerable leak of E proteins such as the large subunit of HSV's ribonucleotide reductase (ICP6) occurs in the absence of ICP4 [48], [49]. To determine if HSV-1 IE proteins were indeed sufficient to promote GFP-tagged ICP0's translocation to the cytoplasm, chemical inhibitors were used to unambiguously separate HSV-1 protein synthesis into its IE and E phases. Vero cells were treated with cycloheximide to block protein translation, and cells were inoculated with 5 pfu per cell of HSV-1 0^+^GFP_105_. After allowing 6 hours for the accumulation of viral IE mRNAs, cycloheximide was removed and cultures were released into medium containing either the mRNA synthesis inhibitor actinomycin D [50], the DNA synthesis inhibitor acyclovir [51] or no inhibitor (vehicle).

**Figure 4:**
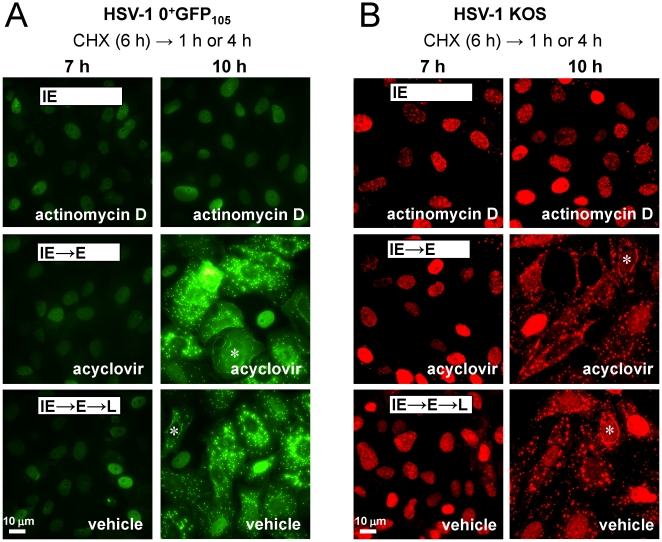
ICP0 translocates to the cytoplasm during the early phase of HSV-1 replication. (A) ICP0^+GFP-105^ protein in Vero cells inoculated with HSV-1 0^+^GFP_105_ following release from a cycloheximide block. Cultures were inoculated with 5 pfu per cell of HSV-1 0^+^GFP_105_, treated with 200 µM cycloheximide from −0.5 to 6 hours p.i., and released into medium containing actinomycin D (10 µg/ml), 300 µM acyclovir, or vehicle (no inhibitor). Cultures were photographed at 7 and 10 hours p.i. (B) ICP0 in HSV-1 KOS-infected Vero cells following release from a cycloheximide block. Cultures were inoculated with 5 pfu per cell of KOS, treated with cycloheximide from −0.5 to 6 hours p.i., released into medium containing actinomycin D, acyclovir, or vehicle, and fixed for immunofluorescent staining at 7 and 10 hours p.i. White asterisks denote cells in which ICP0 was observed in a linear pattern encircling the nucleus of HSV-1 infected cells. The scale bar denotes a distance of 10 µm.

In HSV-1 0^+^GFP_105_-infected cells released into medium containing actinomycin D, ICP0^+GFP-105^ remained in the nuclei of >90% of cells at 1 and 4 hours post-release from a cycloheximide block (Fig. 4A). Thus, translocation of ICP0^+GFP-105^ failed to occur when only viral IE proteins were efficiently expressed. When HSV-1 0^+^GFP_105_-infected cells were released into medium containing acyclovir (IE and E proteins expressed), ICP0^+GFP-105^ was initially observed in nuclei, but translocated to the cytoplasm of most cells by 4 hours post-release (Fig. 4A). Likewise, when cycloheximide treatment was followed with vehicle, ICP0^+GFP-105^ translocated to the cytoplasm with similar kinetics (Fig. 4A). Parallel tests with HSV-1 0^+^GFP_12_, 0^+^GFP_24_, and 0^+^GFP_24_-Δ4 yielded equivalent results (not shown). These results suggested that the nuclear-to-cytoplasmic translocation of ICP0^+GFP-105^ occurred during the E phase of HSV-1 replication.

To determine if these results were relevant to wild-type ICP0, similar tests were performed with wild-type HSV-1 strain KOS [52]. When cycloheximide treatment was followed with actinomycin D, wild-type ICP0 was retained in the nuclei of KOS-infected cells at 4 hours post-release (Fig. 4B). In contrast, when cycloheximide treatment was followed with acyclovir or vehicle, wild-type ICP0 translocated to the cytoplasm by 4 hours post-release and was observed in globular and linear structures that encircled the nuclei of >90% of KOS-infected cells (Fig. 4B). Therefore, both GFP-tagged ICP0 and wild-type ICP0 were predominantly nuclear during the IE phase of viral infection, but rapidly translocated to the cytoplasm when viral E protein synthesis was allowed to occur.

### ICP0^+GFP-105^ disperses linear structures in the cytoplasm of HSV-1 infected cells

The cytoplasmic structures in which ICP0 accumulated did not appear to be mitochondria, lysosomes, Golgi apparatus, or endoplasmic reticulum based upon the failure of ICP0 to co-localize with MitoTracker dye [53], LysoTracker dye [54], Golgi marker β-COP [55], or the endoplasmic reticulum marker calreticulin [56]. Moreover, in early attempts to isolate the fluorescent-ICP0 labeled cytoplasmic bodies by density gradient sedimentation, it was noted that the bodies rapidly dispersed upon homogenization of cells in ice-cold buffer (not shown). To gather further clues about the nature of these structures, live-cell imaging was used to study the dynamics of accumulation of ICP0^+GFP-105^ in the cytoplasm of HSV-1 0^+^GFP_105_-infected cells.

Cycloheximide was used to alleviate ICP4-dependent repression of the *ICP0^GFP^* gene, such that ICP0^+GFP-105^ mRNA could accumulate to high levels and be synchronously translated upon cycloheximide release [9]. At 1 hour post-release, ICP0^+GFP-105^ was observed exclusively in the nuclei of HSV-1 0^+^GFP_105_-infected cells (Table 1, Fig. S1A). Between 2 and 4 hours post-release, ICP0^+GFP-105^ had partially or completely translocated to the cytoplasm of >90% of HSV-1 0^+^GFP_105_-infected cells (Table 1, Fig. S1A). At 4 hours post-release, ICP0^+GFP-105^ was observed in linear cytoplasmic structures in 16±1% of 0^+^GFP_105_-infected cells (Table 1, Fig. S1A). However, by 6 hours post-release, ICP0^+GFP-105^ was rarely observed in linear cytoplasmic structures (Table 1, Fig. S1A).

**Table 1:** Subcellular distribution of ICP0^GFP^ in HSV-1 infected cells at times after release from a cycloheximide block^a^.

Virus	Hours	Subcellular distribution b		Cytoplasmic pattern e	
		ICP0^GFP^ only in nucleus	ICP0^GFP^ in cytoplasm	ICP0^GFP^ in lines	ICP0^GFP^ in globules
HSV-1 0^+^GFP_105_	1	100±0^c^	0±0^d^	0±0^f^	0±0^g^
	2	9±1	91±1	2±0	98±0
	3	2±0	98±0	10±1	90±1
	4	2±0	98±0	16±1	77±1
	5	2±0	98±0	11±1	81±1
	6	1±0	99±0	1±0	87±1
HSV-1 0ΔRING	1	100±0	0±0	0±0	0±0
	2	40±2	60±2	96±0	4±0
	3	39±1	61±1	97±0	3±0
	4	34±1	66±1	99±0	1±0
	5	26±1	74±1	99±0	1±0
	6	22±1	78±1	99±0	1±0

a Vero cells were inoculated with 5 pfu per cell of HSV-1 0^+^GFP_105_ or HSV-1 0ΔRING in the presence of 200 µM cycloheximide from −0.5 to 10 hours p.i., and were released into medium containing no drugs. Cultures were photographed between 1 and 6 hours post-release to record the position of all nuclei (visualized with Hoechst 33342) and the subcellular localization of ICP0^+GFP-105^ or ICP0^ΔRING^. Representative photographs that formed the basis for the data presented above are shown in Fig. S1.

b Frequency of cells in which ICP0^+GFP-105^ or ICP0^ΔRING^ was observed exclusively in the nucleus (left column) versus cells in which proteins were observed in either the cytoplasm and nucleus or just in the cytoplasm (right column).

c Frequency of cells (mean ± sem) in which ICP0^+GFP-105^ or ICP0^ΔRING^ was observed exclusively in the nucleus. The frequency reported at each time point was calculated as 100×number of cells in which ICP0^GFP^ was observed solely in the nucleus÷number of ICP0^GFP+^ cells. Each value is based on n = 4 independent cultures, and each estimate of frequency was based on n = 400 Hoechst^+^ cells. Between 4 and 6 hours post-release, 95±1% of cells in HSV-1 inoculated cultures were positive for the ICP0^+GFP-105^ or ICP0^ΔRING^ proteins.

d Frequency of cells (mean ± sem) in which ICP0^+GFP-105^ or ICP0^ΔRING^ was observed in the cytoplasm and nucleus, or just in the cytoplasm. Frequencies were calculated by the method described in footnote c.

e Frequency of ICP0^+GFP-105^ or ICP0^ΔRING^ accumulating in linear arrays (left column) versus globular patterns (right column) in the cytoplasm of HSV-1 infected cells.

f Frequency of cells (mean ± sem) in which ICP0^+GFP-105^ or ICP0^ΔRING^ were observed in linear cytoplasmic arrays that encircled Hoechst^+^ nuclei. The presence of one or more linear arrays of ICP0^GFP^ was the basis for scoring a cell as having “ICP0^GFP^ in lines.” The frequency reported at each time point was calculated as 100×number of cells containing linear arrays of ICP0^GFP^ protein÷number of cells in which ICP0^GFP^ was evident in the cytoplasm. Each value is based on n = 4 independent cultures, and each estimate of frequency was based on n = 400 Hoechst^+^ cells.

g Frequency of cells (mean ± sem) in which ICP0^+GFP-105^ or ICP0^ΔRING^ were observed in globular, cytoplasmic bodies, which was calculated as described in footnote f.

We considered the possibility that ICP0^+GFP-105^ might trigger the dispersal of cytoplasmic structures in much the same manner that ICP0 triggers the dispersal of PML nuclear bodies [13], [14]. To test this hypothesis, a recombinant virus was constructed, HSV-1 0ΔRING, which encoded a GFP-tagged ICP0^ΔRING^ protein that was deleted of amino acids 105–221, and thus lacked ICP0's RING-finger domain [10]. When cycloheximide-release experiments were performed in cells inoculated with HSV-1 0ΔRING, the ICP0^ΔRING^ protein accumulated in linear cytoplasmic structures between 2 and 6 hours post-release (Table 1, Fig. S1B). Intriguingly, ICP0^ΔRING^ was rarely observed in globular bodies, but rather the protein was observed almost exclusively in linear cytoplasmic structures (Table 1, Fig. S1B). These findings suggested that ICP0^ΔRING^ stably accumulated in linear cytoplasmic structures, whereas ICP0^+GFP-105^ triggered the dispersal of these structures upon accumulating at these sites.

**Figure 5:**
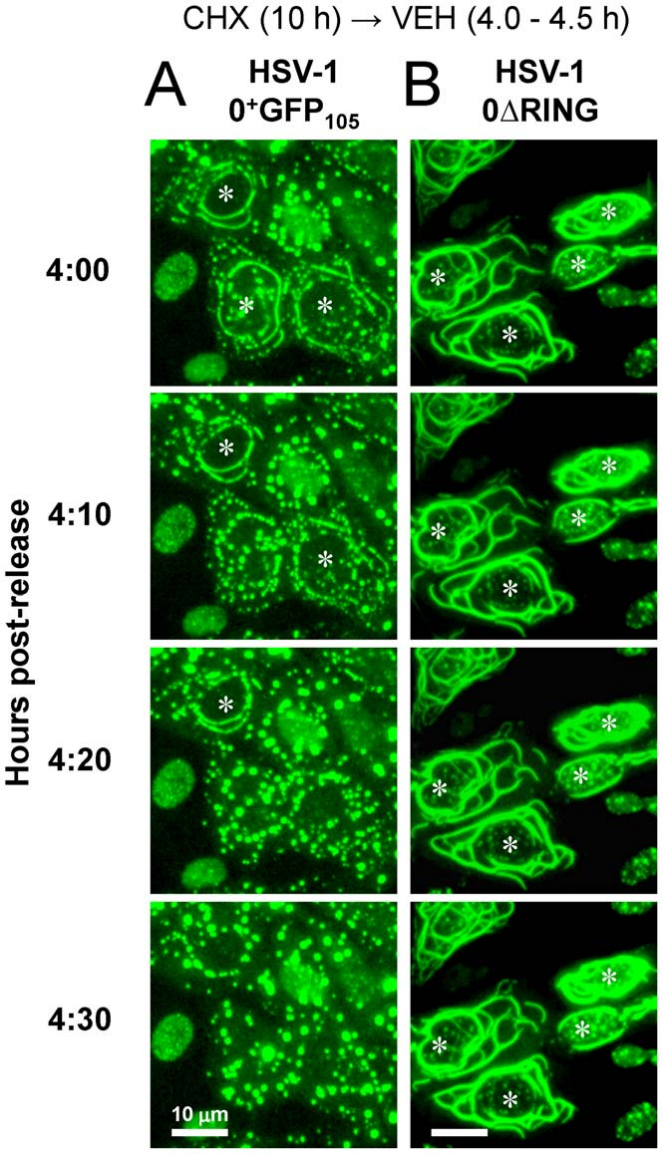
ICP0^+GFP-105^ disperses linear cytoplasmic structures in a RING-finger-dependent manner. Vero cells were inoculated with 5 pfu per cell of (A) HSV-1 0^+^GFP_105_ or (B) HSV-1 0ΔRING in the presence of 200 µM cycloheximide from −0.5 to 10 hours p.i., and were released into medium containing no drugs. Between 4.0 and 4.5 hours post-release, time-lapse photographs were collected of the ICP0^+GFP-105^ or ICP0^ΔRING^ proteins that encircled the nucleus of HSV-1 infected cells. The scale bar denotes a distance of 10 µm.

To test this hypothesis, live-cell imaging was used to track the fate of linear structures in which ICP0^+GFP-105^ accumulated. Cells were inoculated with HSV-1 0^+^GFP_105_ and time-lapse photography was conducted between 4.0 and 4.5 hours post-release from a cycloheximide block. Linear, cytoplasmic arrays of ICP0^+GFP-105^ that initially encircled the nucleus were found to be unstable, and dispersed into smaller, globular bodies over a 10- to 20-minute time frame (Fig. 5A, Video S2). In contrast, experiments with HSV-1 0ΔRING demonstrated that the ICP0^ΔRING^ protein stably accumulated in linear cytoplasmic structures which grew visibly in length and thickness between 4.0 and 4.5 hours post-release (Fig. 5B, Video S3). This trend continued until 6 hours post-release, at which time the experiment was terminated (not shown). Therefore, ICP0^+GFP-105^ translocated to the cytoplasm, accumulated in linear cytoplasmic structures, and then triggered the dispersal of these structures in a RING-finger-dependent manner.

### ICP0 co-localizes with disrupted microtubule networks in HSV-1 infected cells

**Figure 6:**
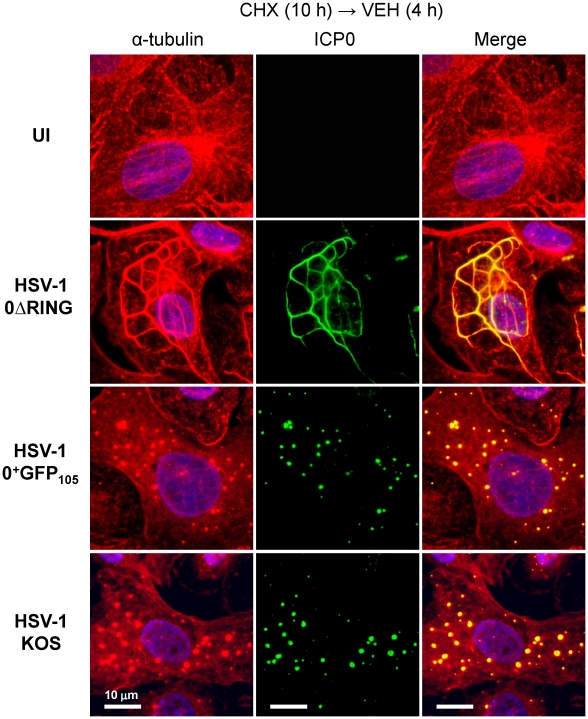
ICP0 co-localizes with reorganized microtubules in HSV-1 infected cells. Immunofluorescent staining of Vero cells that were uninfected (no virus) or that were inoculated with 5 pfu per cell of HSV-1 0ΔRING, HSV-1 0^+^GFP_105,_ or wild-type HSV-1 strain KOS. Cells were inoculated in the presence of 200 µM cycloheximide from −0.5 to 10 hours p.i., and were released into medium containing no drugs. At 4 hours post-release, uninfected cells and KOS-infected cells were fixed and stained with antibodies against α-tubulin (rabbit IgG, Alexa Fluor 594) and ICP0 (mouse IgG, fluorescein). Nuclei were counterstained with the DNA-binding dye Hoechst 33342. HSV-1 0ΔRING and 0^+^GFP_105_-infected cells were fixed and stained for α-tubulin, and the ICP0^ΔRING^ and ICP0^+GFP-105^ proteins were visualized using their GFP fluorophores. The scale bar denotes a distance of 10 µm.

The linear cytoplasmic structures in which ICP0^+GFP-105^ transiently accumulated were reminiscent of microtubule bundles formed by HSV-1's VP22 protein [36]. Therefore, we considered the possibility that ICP0^+GFP-105^ and ICP0^ΔRING^ proteins might associate with host cell microtubules. To test this hypothesis, cycloheximide-release experiments were performed in cells inoculated with HSV-1 0ΔRING, HSV-1 0^+^GFP_105_, or wild-type HSV-1 KOS. At 4 hours post-release, cells were fixed and immunofluorescently stained for α-tubulin and ICP0. In uninfected Vero cells, α-tubulin staining revealed a normal network of microtubules that radiated from the microtubule-organizing center at the periphery of the nucleus (Fig. 6). In cells inoculated with HSV-1 0ΔRING, α-tubulin staining revealed extensive thickening of microtubules into elongated bundles that circumscribed the nucleus, and co-localized with the ICP0^ΔRING^ protein (Fig. 6). In cells inoculated with HSV-1 0^+^GFP_105_, α-tubulin staining revealed that the microtubule network had dispersed into small α-tubulin^+^, globular bodies that co-localized with ICP0^+GFP-105^ (Fig. 6). In cells inoculated with wild-type HSV-1 KOS, the host cell microtubule network was also disrupted, and α-tubulin was dispersed into α-tubulin^+^ globular bodies that co-localized with ICP0 (Fig. 6). Although the ICP0^ΔRING^ protein bundled microtubules, it failed to trigger their dispersal into globular bodies (Fig. 6). These results raised the possibility that HSV's E3 ligase, ICP0, might contribute to microtubule disassembly in HSV-infected cells [35]. Further experiments were conducted to test the validity of this hypothesis.

### ICP0^ΔRING^ protein accumulates in linear structures that are nocodazole-sensitive

If microtubules did indeed provide the underlying scaffold on which ICP0^ΔRING^ protein accumulated in linear arrays (Fig. 5, 6), then inhibition of microtubule polymerization with nocodazole [57] should recapitulate the effect of ICP0's RING finger domain. Specifically, nocodazole treatment, like wild-type ICP0, should trigger microtubule disassembly and cause linear accumulations of ICP0^ΔRING^ protein to disperse into smaller bodies. To test this prediction, cells were infected with HSV-1 0ΔRING in the presence of cycloheximide for 10 hours, and were released into medium containing no inhibitor. Four hours later, the stability of linear arrays of ICP0^ΔRING^ protein was compared in the presence or absence of nocodazole.

**Figure 7:**
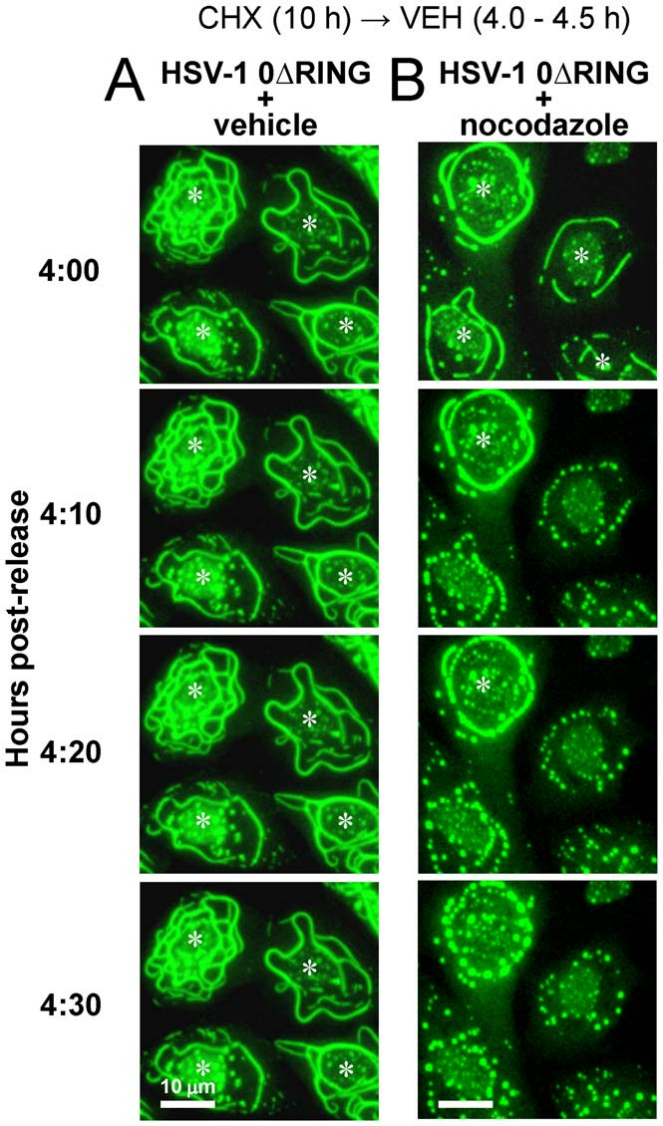
ICP0^ΔRING^ accumulates in linear structures that are nocodazole-sensitive. Vero cells were inoculated with 5 pfu per cell of HSV-1 0ΔRING in the presence of 200 µM cycloheximide from −0.5 to 10 hours p.i., and were released into medium containing no drugs. At 4 hours post-release, a representative field of view was chosen, and cells were treated with (A) vehicle or (B) 10 µg/ml nocodazole. Between 4.0 and 4.5 hours after cycloheximide release, time-lapse photographs were captured at 10-minute intervals. White asterisks denote cells in which ICP0^ΔRING^ was observed in a continuous, linear pattern encircling the nucleus of HSV-1 infected cells. The scale bar denotes a distance of 10 µm.

In the absence of nocodazole, ICP0^ΔRING^ protein stably accumulated in linear structures that encircled the nucleus of HSV-1 0ΔRING-infected cells between 4.0 and 4.5 hours post-release (Fig. 7A, Video S3). In contrast, nocodazole treatment caused linear accumulations of ICP0^ΔRING^ protein to disperse into small globular bodies within 10 to 30 minutes, and the time required for dispersal varied in proportion to the thickness of each bundle (Fig. 7B, Video S4). Nocodazole-induced, ICP0^ΔRING+^ globular bodies were identical in appearance to α-tubulin^+^ globular bodies that co-localized with ICP0 and ICP0^+GFP-105^ (Fig. 6). Independent tests verified that nocodazole-induced, ICP0^ΔRING+^ globular bodies were also α-tubulin^+^ (not shown).

These observations indicated that the linear cytoplasmic structures in which ICP0^ΔRING^ protein accumulated were indeed microtubule bundles. This finding was also consistent with our prior observation that ICP0^+GFP-105^-containing bodies rapidly dispersed upon homogenization in ice-cold buffer; a condition known to cause depolymerization of microtubules [58].

### ICP0 is necessary for efficient dispersal of microtubules in HSV-1 infected cells

**Figure 8:**
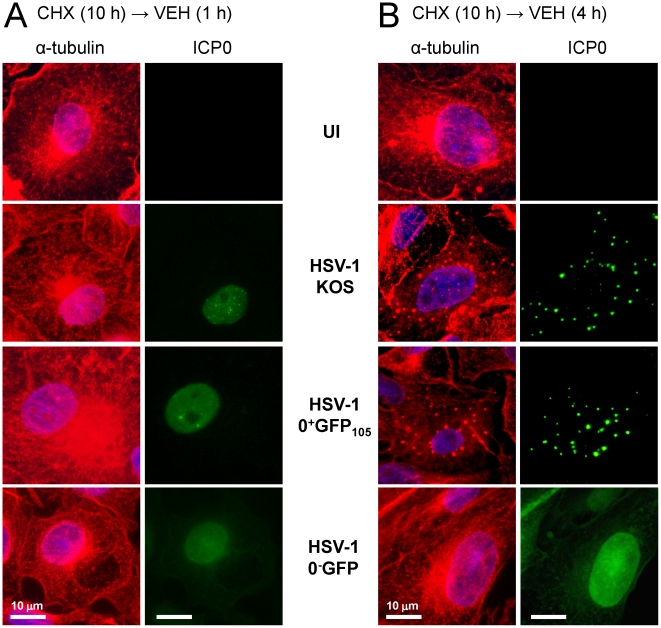
ICP0 is necessary for dispersal of microtubule networks in HSV-1 infected cells. Vero cells were uninfected or were inoculated with 5 pfu per cell of HSV-1 strain KOS, HSV-1 0^+^GFP_105,_ or HSV-1 0^−^GFP in the presence of 200 µM cycloheximide from −0.5 to 10 hours p.i., and were released into medium containing no drugs until the time of fixation. At (A) 1 hour post-release or (B) 4 hours post-release, uninfected and KOS-infected cells were fixed and stained with antibodies against α-tubulin (rabbit IgG) and ICP0 (mAb H1083). HSV-1 0^+^GFP_105_ or 0^−^GFP-infected cells were fixed and stained for α-tubulin, and ICP0^+GFP-105^ and ICP0^−GFP^ proteins were visualized using their GFP fluorophores. Nuclei were counterstained with the DNA-binding dye Hoechst 33342. The scale bar denotes a distance of 10 µm.

It is well established that the host cell microtubule network is disrupted during the course of HSV-1 infection [35], [59], [60]. However, it is unclear what process triggers microtubule reorganization in HSV-1 infected cells. We questioned whether this event might be ICP0-dependent. To test this hypothesis, α-tubulin and ICP0 staining were compared in Vero cells that were uninfected (UI) or were inoculated with HSV-1 KOS, HSV-1 0^+^GFP_105_, or an *ICP0* ^−^ null virus, HSV-1 0^−^GFP. Cells were inoculated with 5 pfu per cell of each virus in the presence of cycloheximide for 10 hours to allow high and equivalent levels of ICP0^+^ and ICP0^−^ mRNAs to accumulate [9]. At 1 and 4 hours post-release, a normal distribution of α-tubulin staining was noted in uninfected Vero cells; specifically, microtubules radiated from the microtubule-organizing center on one side of the nucleus (Fig. 8A). At 1 hour post-release, ICP0 and ICP0^+GFP-105^ were observed in the nuclei of cells infected with HSV-1 KOS and 0^+^GFP_105_ (Fig. 8A). Importantly, α-tubulin staining was always normal when ICP0 or ICP0^+GFP-105^ were confined to the nucleus (Fig. 8A). However, at 4 hours post-release, ICP0 and ICP0^+GFP-105^ had translocated to the cytoplasm and this event coincided with dispersal of microtubules in KOS- and 0^+^GFP_105_-infected cells, respectively (Fig. 8B). Specifically, microtubule-organizing centers were no longer discernible, and α-tubulin was dispersed into globular bodies that co-localized with ICP0 or ICP0^+GFP-105^ (Fig. 8B). In contrast, at 1 and 4 hours post-release, cells inoculated with HSV-1 0^−^GFP retained an intact microtubule network that radiated from a perinuclear microtubule-organizing center (Fig. 8A, 8B). Likewise, at 6 and 8 hours post-release, microtubule networks remained intact in HSV-1 0^−^GFP-infected cells (Fig. S2). Therefore, synthesis of ICP0 appeared to be necessary for the efficient dismantling of microtubule networks that normally occurs in HSV-1 infected cells [35].

### ICP0 is sufficient to dismantle microtubule networks in transfected cells

The HSV-1 proteins US11 [61], UL34 [62], VP26 [63] and VP22 [36] interact with host cell microtubules. Thus, ICP0 might be one of many HSV proteins that is required to disperse the intracellular ‘freeway system’ that is the cell's microtubule network [64]. Alternatively, synthesis of ICP0 alone might be sufficient to disperse cellular microtubules. To differentiate between these possibilities, Vero cells were transfected with pICP0, p0^+^GFP_105_, or p0ΔRING to determine if synthesis of ICP0 proteins affected the subcellular distribution of α-tubulin, one of the principal structural subunits of the hollow, polymeric tubes known as microtubules [65].

**Figure 9:**
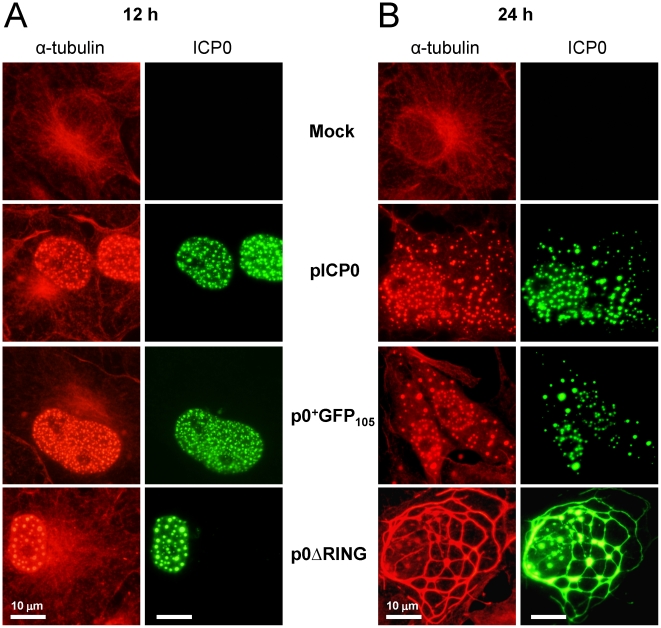
ICP0 is sufficient to trigger dispersal of host cell microtubule networks. Vero cells were mock-transfected or were transfected with a VP16-expressing plasmid and pICP0, p0^+^GFP_105,_ or p0ΔRING. At (A) 12 hours post-transfection or (B) 24 hours post-transfection, mock- and pICP0-transfected cells were fixed and stained with antibodies against α-tubulin (rabbit IgG) and ICP0 (mAb H1083). Cells transfected with p0^+^GFP_105_ or p0ΔRING were fixed and stained to visualize α-tubulin, and the ICP0^ΔRING^ and ICP0^+GFP-105^ proteins were visualized using their GFP fluorophores. The scale bar denotes a distance of 10 µm.

A normal distribution of α-tubulin staining was noted in mock-transfected Vero cells at 12 and 24 hours post-transfection. Specifically, microtubules radiated from the organizing center on one side of the nucleus, and α-tubulin staining was not observed in the nuclei of mock-transfected Vero cells (Fig. 9A, 9B). Consistent with prior results (Fig. 2A–2C), wild-type ICP0 and ICP0^+GFP-105^ were observed in the nuclei of cells transfected with pICP0 and p0^+^GFP_105_ at 12 hours post-transfection (Fig. 9A). At this early time, a normal distribution of α-tubulin staining was noted in the cytoplasm, but to our surprise significant amounts of α-tubulin were present in the nuclei and co-localized with wild-type ICP0 and ICP0^+GFP-105^ (Fig. 9A, Fig. S3). Control staining with individual antibodies verified that the appearance of co-localization was not an unintended consequence of bleedover between the red and green channels (not shown). In cells transfected with p0ΔRING, the ICP0^ΔRING^ protein was also observed in the nuclei of cells at 12 hours post-transfection, and a normal distribution of α-tubulin staining was noted in the cytoplasm. However, large amounts of α-tubulin were present in the nuclei of transfected cells, and α-tubulin co-localized with the ICP0^ΔRING^ protein (Fig. 9A, Fig. S3).

At 24 hours post-transfection, wild-type ICP0 and ICP0^+GFP-105^ were observed in the nuclei and cytoplasm of cells transfected with pICP0 and p0^+^GFP_105_, respectively (Fig. 9B). Translocation of ICP0 or ICP0^+GFP-105^ to the cytoplasm consistently correlated with ***1.*** dissolution of the host cell microtubule-organizing center and microtubule network, and ***2.*** co-localization of α-tubulin with ICP0 or ICP0^+GFP-105^ in small, globular bodies in the cytoplasm (Fig. 9B; Fig. S3). Likewise, 24 hours after transfection with p0ΔRING, ICP0^ΔRING^ protein co-localized with α-tubulin in elongated microtubule bundles (Fig. 9; Fig. S3). Therefore, synthesis of the ICP0^ΔRING^ protein was sufficient to trigger bundling, but not dispersal of microtubules. In contrast, wild-type ICP0 was sufficient to trigger a complete dispersal of host cell microtubules.

### ICP0 translocation and microtubule dispersal in a single HSV-1 plaque

**Figure 10:**
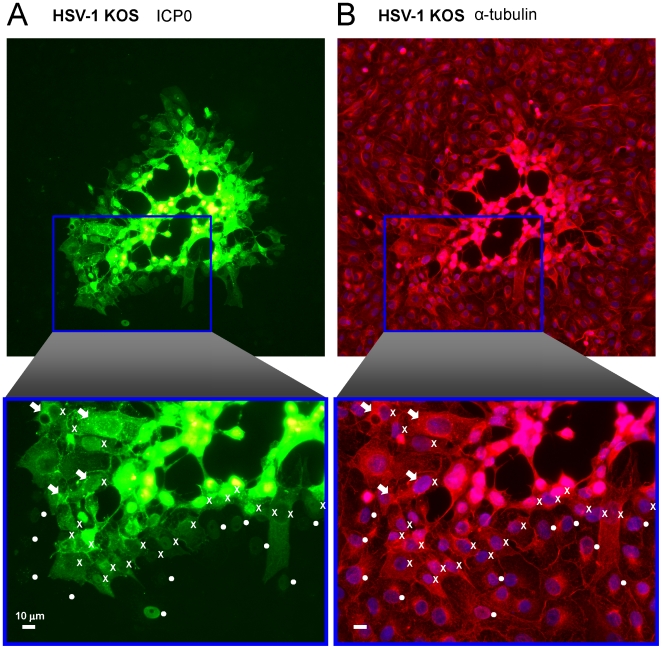
Translocation of ICP0 and disruption of host cell microtubules in a single HSV plaque. Vero cells were inoculated with wild-type HSV-1 KOS virus at an MOI of 0.0001 pfu per cell, and cells were fixed and stained at 40 hours p.i. using antibodies against (A) ICP0 and (B) α-tubulin. Nuclei were counterstained with the DNA-binding dye Hoechst 33342. Cells in which ICP0 was observed solely in the nucleus are denoted by a circle (•) to the right of the nucleus. Cells in which ICP0 was prominent in the cytoplasm are denoted with an ‘X’ to the right of the nucleus. White arrows denote cells in which α-tubulin was not only dispersed, but also co-localized with ICP0. The scale bar denotes a distance of 10 µm.

High MOIs and cycloheximide-release experiments allowed robust visualization of ICP0 and co-localization of ICP0 with dispersed α-tubulin. These tests left unaddressed the question of whether or not ICP0 actually translocates and/or dismantles microtubule networks during the normal progression of HSV-1 infection. To address this issue, Vero cells were inoculated with wild-type virus at an MOI of 0.0001 pfu per cell, and the distribution of ICP0 and α-tubulin was analyzed in isolated HSV-1 plaques at 40 hours p.i. A single, representative plaque is considered to illustrate our findings (Fig. 10, Fig. S4).

With the enhanced sensitivity of immunofluorescent staining (versus direct imaging of GFP fluorescence, Fig. 2), low levels of ICP0 were detectable in the nuclei of cells at the outermost, advancing edge of HSV-1 plaques (• symbols, Fig. 10A, Fig. S4A). In those HSV-1 infected cells in which ICP0 was observed solely in the nucleus, the perinuclear pattern of microtubule staining remained normal (• symbols, Fig. 10B, Fig. S4B). Behind this advancing front, a second row of HSV-1 infected cells was observed in which ICP0 was far more abundant and predominantly localized to the cytoplasm of HSV-1 infected cells (X symbols, Fig. 10A, Fig. S4A). When ICP0 accumulated in the cytoplasm of HSV-1 infected cells, gross reorganization of the cellular microtubule network was observed (X symbols, Fig. 10B, Fig. S4B). Moreover, α-tubulin was frequently observed reorganized into linear structures or globular bodies that co-localized with wild-type ICP0 (white arrows, Fig. 10, Fig. S4). Therefore, we conclude that regardless of MOI, a nuclear-to-cytoplasmic translocation of ICP0 routinely occurs in HSV-1 infected cells, and the timing of ICP0's translocation to the cytoplasm coincides with a massive reorganization of the host cell's microtubule network.

## Discussion

### ICP0 serves distinct roles in the nucleus and cytoplasm of HSV-infected cells

Studies of ICP0 have focused on the protein's role in the nucleus [18], [30], [31], [32]. Cytoplasmic ICP0 has been repeatedly observed [21], [22], [23], [24], [25], but its significance remains obscure for two reasons. First, the kinetics of ICP0's nuclear-to-cytoplasmic translocation has been poorly defined. Thus, it has not been apparent that ICP0's function in the cytoplasm is kinetically delayed and is only manifest once ICP0 fulfills its IE function in the nucleus [7], [8], [9]. Second, the role that ICP0 fulfills in the cytoplasm of HSV-infected cells has not been clearly articulated. The results of the current study address these gaps in knowledge.

The results of the current study demonstrate that once E proteins accumulate, ICP0 translocates to the cytoplasm (Fig. 4) and there dismantles the host cell's microtubule network. Five observations support this conclusion: ***1.*** Microtubule dispersal is ICP0-dependent in HSV-infected cells (Fig. 8); ***2.*** Synthesis of ICP0 is sufficient to trigger microtubule dispersal (Fig. 9); ***3.*** Dispersal of microtubule bundles is dependent upon ICP0's RING finger domain (Fig. 6); ***4.*** The timing of microtubule dispersal coincides with ICP0's translocation to the cytoplasm (Fig. 8); and ***5.*** dispersed α-tubulin co-localizes with ICP0 (Fig. S3).

### GFP-tagged ICP0 yields new insights into the biology of ICP0

HSV-1 viruses that encoded chimeric ICP0^+GFP^ proteins formed plaques with ∼66% efficiency relative to wild-type HSV-1 (Fig. 1E). Using this new tool, we were able to observe GFP-tagged ICP0 in the act of bundling and/or dispersing microtubules in the cytoplasm of HSV-infected cells (Videos S2, S3). These findings would be difficult to ascertain from analysis of fixed cells because ICP0's cytoplasmic pattern is pleomorphic in HSV-infected cells. However, direct observation of a single HSV-infected cell in real time revealed the fluid process by which GFP-tagged ICP0 triggered the disassembly of subcellular structures that proved to be bundled microtubules (Figs. 5 and 6; Table 1; Video S2).

### Virus-induced reorganization of host cell microtubules

Many animal and plant viruses use host cell microtubule networks during their replication cycle [39], [40], [41]. For example, movement protein of tobacco mosaic virus [66] and transmission factor of cauliflower mosaic virus [67] modify microtubules in plant cells. The human immunodeficiency virus Rev protein forms dimers that bind microtubule ends, and inhibit their polymerization [68]. The 3C protease of foot-and-mouth disease virus cleaves microtubule-associated protein 4, and thus excludes microtubules from cytoplasmic replication compartments [38], [69]. Expression of Epstein-Barr virus's LMP-1 protein or SV40's large T antigen causes formation of aberrant microtubule structures via the respective modulation of microtubule-stabilizing proteins RASSF1 A [70] and TACC2 [39]. While many viruses modify the microtubule network during their replication cycles, the functional significance of these interactions is often unclear. Thus, there is no clear virological precedent that explains why HSV should encode a protein, ICP0, that dismantles the microtubule network.

### ICP0: a viral E3 ligase that orchestrates microtubule disassembly

The current study provides the first example of a viral E3 ligase that triggers microtubule disassembly. The genomes of at least 16 α-herpesviruses encode RING-finger E3 ligases, and these proteins constitute a family of ICP0-like proteins that are functional homologs of one another [45]. Therefore, it is reasonable to expect that the ICP0-like proteins of all α-herpesviruses should likewise fulfill dual roles in the nucleus and cytoplasm of virus-infected cells [9], and thus should trigger disassembly of microtubule networks upon their translocation to the cytoplasm. Further testing will be required to validate this prediction.

The α-herpesviruses are not alone in their use of E3 ligases as regulatory molecules. The Rta protein of Kaposi's sarcoma herpesvirus possesses intrinsic E3 ligase activity despite the absence of a canonical RING finger domain [71]. Smaller RNA and DNA viruses exploit ubiquitination as a regulatory mechanism by encoding adapter proteins such as adenovirus E1b, papillomavirus E6 and E7, or paramyxovirus V proteins that redirect cellular E3 ligases to substrates that benefit the virus [71], [72], [73], [74], [75]. It will be of interest to determine in coming years if any of these other viral E3 ligases reorganize host cell microtubule networks.

Many cellular E3 ligases play a prominent role in coordinating the complex events that transpire during mitosis, and which include disassembly of the microtubule network [43]. For example, cullin 3 is an E3 ligase that regulates the activity of a microtubule-severing protein known as katanin (i.e., ‘sword’ in Japanese; Ref. [43], [44], [76]). Given the propensity of viruses to steal useful cellular functions [71], [77], our results raise the possibility that ICP0 may be functionally homologous to cellular E3 ligases that influence cell-cycle progression by regulating the stability of microtubule networks [43]. Further investigation will be required to explore this important hypothesis.

### Is α-tubulin a substrate of ICP0's E3 ligase activity?

The discovery of ICP0's E3 ligase activity by Everett and colleagues [10], [78] arose from a need to explain why PML nuclear bodies were dispersed shortly after ICP0 arrived in these sub-nuclear structures [13], [14], [15]. Although it seemed likely that PML would prove to be a direct substrate of ICP0's E3 ligase activity, the development of a robust *in vitro* assay for ICP0's E3 ligase activity demonstrated that this was not the case [10], [17].

It is possible that α-tubulin may be a direct substrate of ICP0's E3 ligase activity, but the history of ICP0 research suggests that such speculation is premature. What should be considered, however, is the extraordinarily robust co-localization of α-tubulin and ICP0. For example, in transfected cells, large amounts of α-tubulin co-localized with ICP0 *in the nucleus*, which is not normally observed in uninfected cells (Fig. 9, Fig. S3). These results suggest that newly synthesized ICP0 may be tethered to α-tubulin so rapidly in the cytoplasm that the ICP0-containing complexes that are imported into the nucleus may also contain sizable amounts of α-tubulin. Moreover, ICP0 co-localized with dispersed α-tubulin^+^ structures for hours after disassembly of the microtubule network (Fig. 6, 8, 9). In contrast, ICP0's nuclear interactions with PML [13], [15] and ICP4 [9], [79] appear to be far more transient in nature. Clearly, further work is needed to define how ICP0's E3 ligase activity triggers microtubule disassembly in host cells, and to define why ICP0's co-localization with α-tubulin appears to be so much more stable than any other cellular protein identified to date.

### ICP0-induced disassembly of microtubules versus G2/M cell cycle arrest

Synthesis of ICP0 causes dividing cells to arrest in the G2/M phase of the cell cycle [18], [19], [20]. Two explanations for this phenomenon have been offered including ICP0-induced degradation of centromere protein-C [18], and ICP0-induced activation of a DNA damage response pathway [80]. The current study suggests a third possibility; ICP0-induced disassembly of microtubules produces the same type of G2/M arrest that is caused by nocodazole [81]. Like, nocodazole, ICP0 triggers the rapid disassembly of microtubule networks in cells. Nocodazole causes cells to arrest in the G2/M phase of the cell cycle by blocking mitotic spindle formation, which is needed to align chromosomes on the metaphase plate prior to their segregation into daughter cells [81]. Likewise, synthesis of ICP0 has nocodazole-like effects on cell division [19], [20]. Specifically, chromosome condensation occurs normally in cells treated with ICP0-expressing viruses [18] or nocodazole [82], but in both cases the condensed chromosomes fail to align on metaphase plates [18], [82]. Further work will be required to test the validity of this interpretation, and determine if ICP0-induced disassembly of microtubules explains why synthesis of ICP0 causes cell division to stall during mitosis.

### Conclusion

In recent years, the list of viruses that interact with cellular microtubules has grown in length, but the general significance of the phenomenon remains unclear. Herpesviruses rely on microtubules as a ‘conveyor belt’ to carry their incoming virions from the cell membrane to the nuclear pore [64], [83]. Therefore, it is conceivable that disruption of this conveyor belt may be critical for virion egress, such that newly formed herpesvirus virions may flow efficiently in the reverse direction, from the nucleus back to the cell membrane. Clearly, further studies will be required to determine precisely why ICP0 translocates to the cytoplasm and destabilizes the host cell microtubule network during the E phase of HSV infection.

## Materials and Methods

### Cells, viruses, and plasmids

Vero cells, ICP0-complementing L7 cells [84], and ICP4-complementing E5 cells [85] were propagated in Dulbecco's Modified Eagle's medium (DMEM) containing 0.15% HCO_3_^−^ supplemented with 5% fetal bovine serum (FBS), penicillin G (100 U/ml), and streptomycin (100 mg/ml), hereafter referred to as “complete DMEM.” ICP4-complementing E5 and ICP0-complementing L7 cell lines were kindly provided by Neal Deluca (University of Pittsburgh; [84], [85]). HSV-1 strain KOS was used as the parent wild-type virus in this study. KOS and all of the HSV-1 recombinant viruses used in this study were propagated in Vero cells, L7 cells, or E5 cells cultured in complete DMEM. KOS-GFP is a recombinant virus derived from HSV-1 strain KOS that expresses GFP from a CMV promoter cassette inserted in the intergenic region at the 3′ ends of the UL26 and UL27 genes [47], [86]. To construct HSV-1 recombinant viruses, a 7.3 kb DNA fragment which encompasses the entire *LAT-ICP0* locus was first subcloned from HSV-1 strain KOS into a pCRII plasmid vector (Invitrogen Corporation, Carlsbad, CA). Mutations were introduced into the *ICP0* gene of this plasmid, as described below.

#### i. p0+GFP12

A *GFP* coding sequence was amplified from plasmid peGFP-N1 (Clontech Laboratories, Mountain View, CA) using PCR primers that added NcoI and Bsu36I restriction sites to the 5′ and 3′ ends of the GFP coding sequence. Following PCR amplification with a high fidelity mixture of thermostable DNA polymerases, the *GFP* sequence was subcloned into NcoI and Bsu36I sites that occur in codons 1 and 11 of the *ICP0* coding sequence. The resulting plasmid, p0^+^GFP_12_, encoded ICP0^+GFP-12^ protein. The genetic identity of this and all *GFP* insertions in the *ICP0* gene was confirmed by DNA sequencing. Homologous recombination between HSV-1 KOS and the p0^+^GFP_12_ plasmid yielded the recombinant virus HSV-1 0^+^GFP_12_.

#### ii. p0+GFP24

The plasmid p0^+^GFP_24_ was created by PCR amplification of three DNA fragments that were ligated in series to create a *GFP* insertion at the 5′ end of exon 2 of the *ICP0* gene (Fig. 1A). Specifically, **1.** the 3′ end of intron 1 of the *ICP0* gene; **2.** a *GFP* coding sequence; and **3.** the 5′ half of exon 2 of the *ICP0* gene were PCR-amplified, ligated together, sequenced, and the resulting BamHI - XhoI fragment was subcloned into the BamHI - XhoI sites in the *ICP0* gene. The resulting plasmid, p0^+^GFP_24_, encoded ICP0^+GFP-24^ protein in which GFP is inserted between amino acids 23 and 24 of ICP0. Homologous recombination between HSV-1 KOS and the p0^+^GFP_24_ plasmid yielded the recombinant virus HSV-1 0^+^GFP_24_.

#### iii. p0-GFP

The plasmid p0^−^GFP gene was created by inserting a polylinker, *GFP* coding sequence, and stop codon derived from the plasmid peGFP-N1 (Clontech Laboratories) into a XhoI site in codon 104 of the *ICP0 gene*. The resulting plasmid, p0^−^GFP, encoded the N-terminal 104 amino acids of ICP0, a 14 amino acid linker, and GFP. Homologous recombination between HSV-1 KOS and the p0^−^GFP plasmid yielded the recombinant virus HSV-1 0^−^GFP.

#### iv. p0+GFP105

The plasmid p0^+^GFP_105_ was derived by subcloning a dsDNA linker containing BsrGI and XhoI sites ( tgtaca agatat ctcgag ) in the 3′ end of the *GFP* coding sequence in p0^−^GFP. Thus, a TAA terminator codon was removed and the *GFP* and *ICP0* coding sequences were placed in the same open-reading frame. The resulting plasmid, p0^+^GFP_105_, encoded ICP0^+GFP-105^ protein in which GFP is inserted between amino acids 104 and 105 of ICP0. Homologous recombination between HSV-1 KOS and p0^+^GFP_105_ yielded the virus HSV-1 0^+^GFP_105_.

#### v. p0ΔRING

The plasmid p0ΔRING was derived by deletion of codons 105–229 of the *ICP0* gene from p0^+^GFP_105_. Homologous recombination between HSV-1 KOS and the p0ΔRING plasmid yielded the recombinant virus HSV-1 0ΔRING.

### Construction of recombinant HSV-1 viruses

Infectious HSV-1 DNA was prepared by a protocol that relies upon dialysis to minimize shearing of genome-length HSV-1 DNA; this is a modification of a protocol that was generously provided by Karen Mossman (McMaster University, Hamilton, Ontario). Five 100 mm dishes of Vero cells (3×10^7^ cells) were inoculated with 5 pfu per cell of HSV-1 strain KOS. After 24 hours, cells were scraped, centrifuged, rinsed with PBS, resuspended in 7.0 ml of 200 mM EDTA pH 8.0, and transferred into a 15 ml conical. Proteinase K (75 µl of 10 mg/ml) and 375 µl of 10% SDS were added to virus-infected cells, and the tube was incubated in a rotisserie oven with slow rotation at 50°C for 16 hours. Proteins were removed by phenol : chloroform extraction, and DNA was transferred into a 0.5–3.0 mL Slide-a-lyzer cassette (10,000 MW cutoff; Pierce Chemical Co., Rockford, IL) and dialyzed against 0.1× standard saline citrate for 24 hours. Following dialysis, infectious HSV-1 DNA was aliquoted and frozen at −80°C.

Recombinant HSV-1 viruses were generated by co-transfection of 2 µg infectious HSV-1 KOS DNA and 1 µg each plasmid into a 60 mm dish containing 8×10^5^ ICP0-complementing L7 cells. After 12 hours, medium was replaced with complete DMEM containing 1% methylcellulose and GFP^+^ plaques were selected on a Nikon TE2000 fluorescent microscope (Nikon Instruments, Lewisville, TX). GFP^+^ recombinant viruses were repeatedly passed in ICP0-complementing L7 cells until a uniform population of viruses was obtained that produced 100% GFP^+^ plaques, at which time Southern blot analysis was used to confirm that the anticipated mutation had been introduced into the *ICP0* gene of HSV-1.

### Southern blot analysis

Vero cell cultures were established at a density of 1.5×10^6^ cells per 100 mm dish and were inoculated with viruses at an MOI of 5 pfu per cell. DNA was harvested from virus-infected cells or uninfected controls at 24 hours p.i. using a standard DNA extraction procedure and Southern blot analysis was performed as previously described [87], [88]. Oligonucleotide probes specific for intron 1 of the *ICP0 gene* (5′-cccctagatgcgtgtgagtaaggggggcctgcgtatgagt-3′) were used to probe restriction fragments of HSV-1 viruses.

### Northern blot analysis of ICP0^GFP^ mRNA

Vero cell cultures were established at a density of 1.5×10^6^ cells per plate in 60 mm dishes. Vero cells were inoculated with viruses at an MOI of 10 pfu per cell. RNA was isolated from each treatment group at 12 hours p.i. using Ultraspec RNA isolation reagent (Biotecx Inc., Houston, TX), and Northern blot analysis was performed as previously described [2], [9]. The oligonucleotide probes used were specific for either exon 3 of ICP0 mRNA (5′-ggagtcgctgatcactatggggtctctgttgtttgcaagg-3′) or the GFP coding sequence (5′-atagacgttgtggctgttgtagttgtactcc-agcttgtgc-3′) [9], [89].

### Western blot analysis of ICP0^GFP^ reporter proteins

Vero cell cultures were established at a density of 3×10^5^ cells per well in 12-well plates, and were infected at an MOI of 10 pfu per cell. After 18 hours, proteins were harvested using mammalian protein extraction reagent (Pierce Chemical Co., Rockford, IL) supplemented with 1 mM dithiothreitol and protease inhibitor cocktail set I (Calbiochem, La Jolla, CA). After heat denaturation, 20 µg of each protein sample and MagicMark™ XP protein MW markers (Invitrogen Corporation, Carlsbad, CA) were resolved in a 10% polyacrylamide gel with a 4% stacking gel, and were transferred to nitrocellulose membranes. Protein blots were blocked in phosphate-buffered saline (PBS) containing 5% nonfat dry milk, and were incubated overnight at 4°C in PBS+0.1% Tween-20+5% nonfat dry milk containing a 1∶1000 dilution of mouse monoclonal H1083 antibody specific for amino acids 395 to 775 of ICP0 [79], [90], [91] (EastCoast Bio, North Berwick, MA) and a 1∶500 dilution of rabbit polyclonal anti-GFP antibody (Clontech Laboratories Inc.). Following incubation with primary antibodies, membranes were washed four times with PBS+0.1% Tween-20 (PBS-T), and were incubated for 1 hour with 1∶20,000 dilution of goat anti-rabbit IgG and goat anti-mouse IgG conjugated, respectively, to the infrared fluorescent dyes IRDye® 680 and IRDye® 800CW (LI-COR Bioscience, Lincoln, NE). Protein blots were washed three times in PBS-T, and were scanned for two-color fluorescence using the Odyssey Infrared imaging system (LI-COR Bioscience). Data were analyzed using Odyssey application software version 3.0.16 (LI-COR Bioscience).

### Immunofluorescent staining in Vero cells

Immunofluorescent staining in Vero cells was performed using an adaptation of a staining protocol that was generously provided by Roger Everett (MRC Virology Unit, Glasgow, Scotland). Glass coverslips were placed on the bottom of 6-well dishes and Vero cells were seeded at a density of 1×10^6^ cells per well. After allowing 8 hours for cell attachment, cultures were inoculated with HSV-1 viruses using the conditions defined in each Figure Legend and Results sub-section. At the indicated time of harvest, glass coverslips were removed from culture wells with fine forceps, and were fixed with PBS containing 1.9% formaldehyde and 2% sucrose for 10 minutes, followed by permeabilization with 90% methanol for 10 minutes. HSV-1 Fc-γ receptors (glycoprotein E-I heterodimers [92]) were blocked along with all other non-specific protein-binding sites by incubating fixed cells in a solution of PBS containing 0.5% fetal bovine serum (FBS), as well as 10 µg/ml each of the γ-globulin fractions of human, donkey, and goat serum (Jackson ImmunoResearch Laboratories, Inc., West Grove, PA). Fixed cells were incubated in PBS+FBS+γ-globulin block solution for 10 minutes, and the same solution was used as the diluent for primary and secondary antibodies. Each protein of interest was labeled by incubating fixed cells in a 1∶1000 dilution of primary antibody for 16 hours. Excess primary antibody was removed by washing with PBS+FBS+γ-globulin block solution, and cells were then incubated for 1 hour in PBS+FBS+γ-globulin block containing a 1∶1000 dilution of each secondary antibody as well as 10 ng/ml of Hoechst 33342 to apply a blue label to the nuclei of cells (Calbiochem, La Jolla, CA). Excess secondary antibody was removed by washing with the PBS+FBS+γ-globulin block solution, and coverslips were mounted on glass slides using FluorSave Reagent (Calbiochem, Gibbstown, NJ). Cells on coverslips were photographed using an Olympus BX41 microscope equipped with Olympus DP70 digital camera (Olympus America, Center Valley, PA). Images of green, red, and blue fluorescence were captured at exposure times of 500, 200, and 20 ms, respectively.

The primary antibodies were mouse α-ICP0 monoclonal antibody H1083 (EastCoast Bio), rabbit anti-α-tubulin polyclonal antibody ab18251 (Abcam, Cambridge, MA), rabbit anti-β-COP polyclonal antibody ab2899 (Abcam, Cambridge, MA), rabbit anti-calreticulin polyclonal antibody ab4 (Abcam, Cambridge, MA). The secondary antibodies were Alexa Fluor 594-conjugated goat α-mouse IgG (Molecular Probes, Eugene, OR), fluorescein-conjugated goat α-mouse IgG (Jackson ImmunoResearch), or DyLight 594-conjugated donkey α-rabbit IgG (Jackson ImmunoResearch).

### Live-cell imaging and video analysis of GFP-tagged ICP0 in HSV-1 infected cells

Live-cell imaging was performed by placing a Nikon TE2000 microscope in a 37°C warm room and culturing Vero cells in HEPES-buffered (pH 7.9) RPMI medium containing 5% fetal bovine serum (Gibco BRL, Gaithersburg MD). Time-lapse photography was made possible by outfitting the microscope with an Olympus DP72 digital camera (Olympus America, Center Valley, PA) and a computer containing Olympus DP2-BSW microscope digital camera software which controlled the operation of a Lambda SC SmartShutter™ control system (Sutter Instrument, Novato, CA). Images were captured at 30-second intervals to create Videos S1, S2, S3, S4, or were captured at 10-minute intervals to create static Figures 5 and 7. Time-lapse videos were set to play at a rate of 5 frames per second (2.5 minutes per second of video), such that the motion of GFP-tagged ICP0 shown in videos occurs 150 times faster than real time.

### Transfection of Vero cells

Vero cells were transfected with plasmid DNA (0.75µg/well in 12-well plates) using Lipofectamine 2000 (Invitrogen Corporation, Carlsbad, CA) in complete DMEM at 37°C. Medium was replaced at 4 hours post-transfection to avoid toxicity associated with the transfection reagent, and cells were incubated at 37°C in complete DMEM until cells were fixed for immunofluorescent staining.

## Supporting Information

Figure S1 ICP0^+GFP-105^ disperses linear cytoplasmic structures in HSV-1 0^+^GFP_105_-infected cells. Vero cells were inoculated with 5 pfu per cell of (A) HSV-1 0^+^GFP_105_ or (B) HSV-1 0ΔRING in the presence of 200 µM cycloheximide from −0.5 to 10 hours p.i., and were released into medium containing no drugs. Photographs of (A) ICP0^+GFP-105^ or (B) ICP0^ΔRING^ between 1 and 6 hours after release from the cycloheximide block. The scale bar denotes a distance of 10 µm.
(6.55 MB TIF)

Figure S2 Microtubule networks remain intact in cells infected with an ICP0^−^ null virus, HSV-1 0^−^GFP. Vero cells were uninfected or were inoculated with 5 pfu per cell of inoculated with HSV-1 0^−^GFP in the presence of 200 µM cycloheximide from −0.5 to 10 hours p.i., and were released into medium containing no drugs. At 4, 6, or 8 hours post-release, cells were fixed and stained with rabbit antibody against α-tubulin and the truncated ICP0^−GFP^ peptide was directly visualized. The scale bar denotes a distance of 10 µm.
(5.81 MB TIF)

Figure S3 Co-localization of ICP0 and α-tubulin in cells transfected with ICP0-expressing plasmids. Merged images of the α-tubulin and ICP0 staining shown in Figure 9 in cells that were mock transfected, or which were transfected with pICP0, p0^+^GFP_105_, or p0ΔRING. The scale bar denotes a distance of 10 µm.
(7.18 MB TIF)

Figure S4 Enlarged image of one portion of an HSV-1 plaque in which ICP0 translocation, α-tubulin dispersal, and co-localization of ICP0 and α-tubulin are observed. Enlarged photographs of (A) ICP0-staining and (B) α-tubulin staining from Figure 10.
(7.30 MB TIF)

Video S1 Rapid movement of ICP0^+GFP-24^-labeled globular bodies in HSV-1 0^+^GFP_24_-Δ4-infected cells. Time-lapse photographs of ICP0^+GFP-24^ protein in a representative field of Vero cells between 6.0 and 6.5 hours after inoculation with 5 pfu per cell of HSV-1 0^+^GFP_24_-Δ4. Cultures were continuously incubated at 37°C before and during time-lapse photography. Photographs were captured at a rate of twice per minute for 30 minutes, and are shown in this video at an elapsed rate that is 150 times faster than normal.
(6.26 MB MOV)

Video S2 ICP0^+GFP-105^ disperses linear cytoplasmic structures in HSV-1 0^+^GFP_105_-infected cells. Time-lapse photographs of ICP0^+GFP-105^ protein in a representative field of Vero cells between 4.0 and 4.5 hours post-release from a cycloheximide block. Vero cells were inoculated with 5 pfu per cell of HSV-1 0^+^GFP_105_ in the presence of 200 µM cycloheximide from −0.5 to 10 hours p.i., and were released into medium containing no drugs. At 4.0 hours post-release, a field of cells was chosen where ICP0^+GFP-105^ was present in linear cytoplasmic structures. Cultures were continuously incubated at 37°C before and during time-lapse photography. Photographs were captured at a rate of twice per minute for 30 minutes, and are shown in this video at an elapsed rate that is 150 times faster than normal.
(7.70 MB MOV)

Video S3 ICP0^ΔRING^ accumulates in linear cytoplasmic structures in HSV-1 0ΔRING-infected cells. Time-lapse photographs of ICP0^ΔRING^ protein in a representative field of Vero cells between 4.0 and 4.5 hours post-release from a cycloheximide block. Vero cells were inoculated with 5 pfu per cell of HSV-1 0ΔRING in the presence of 200 µM cycloheximide from −0.5 to 10 hours p.i., and were released into medium containing no drugs. At 4.0 hours post-release, a field of cells was chosen where ICP0^ΔRING^ was present in linear cytoplasmic structures. Cultures were continuously incubated at 37°C before and during time-lapse photography. Photographs were captured at a rate of twice per minute for 30 minutes, and are shown in this video at an elapsed rate that is 150 times faster than normal.
(9.37 MB MOV)

Video S4 Bundles of ICP0^ΔRING^ disperse following nocodazole treatment of HSV-1 0ΔRING-infected cells. Vero cells were inoculated with 5 pfu per cell of HSV-1 0ΔRING in the presence of 200 µM cycloheximide from −0.5 to 10 hours p.i., and were released into medium containing no drugs. At 4.0 hours post-release, a field of cells was chosen and a 3× solution of nocodazole was added to culture medium to achieve a final concentration of 10 µg/ml. Time-lapse photography of ICP0^ΔRING^ protein was commenced 1 minute later. Cultures were continuously incubated at 37°C before and during time-lapse photography. Photographs were captured at a rate of twice per minute for 30 minutes, and are shown in this video at an elapsed rate that is 150 times faster than normal.
(5.42 MB MOV)
