# Supplementary figures and images for: ICP0 Dismantles Microtubule Networks in Herpes Simplex Virus-Infected Cells
Source: PLoS One. 2010 Jun 8;5(6):e10975. doi: 10.1371/journal.pone.0010975 (PMC2882321; doi:10.1371/journal.pone.0010975)

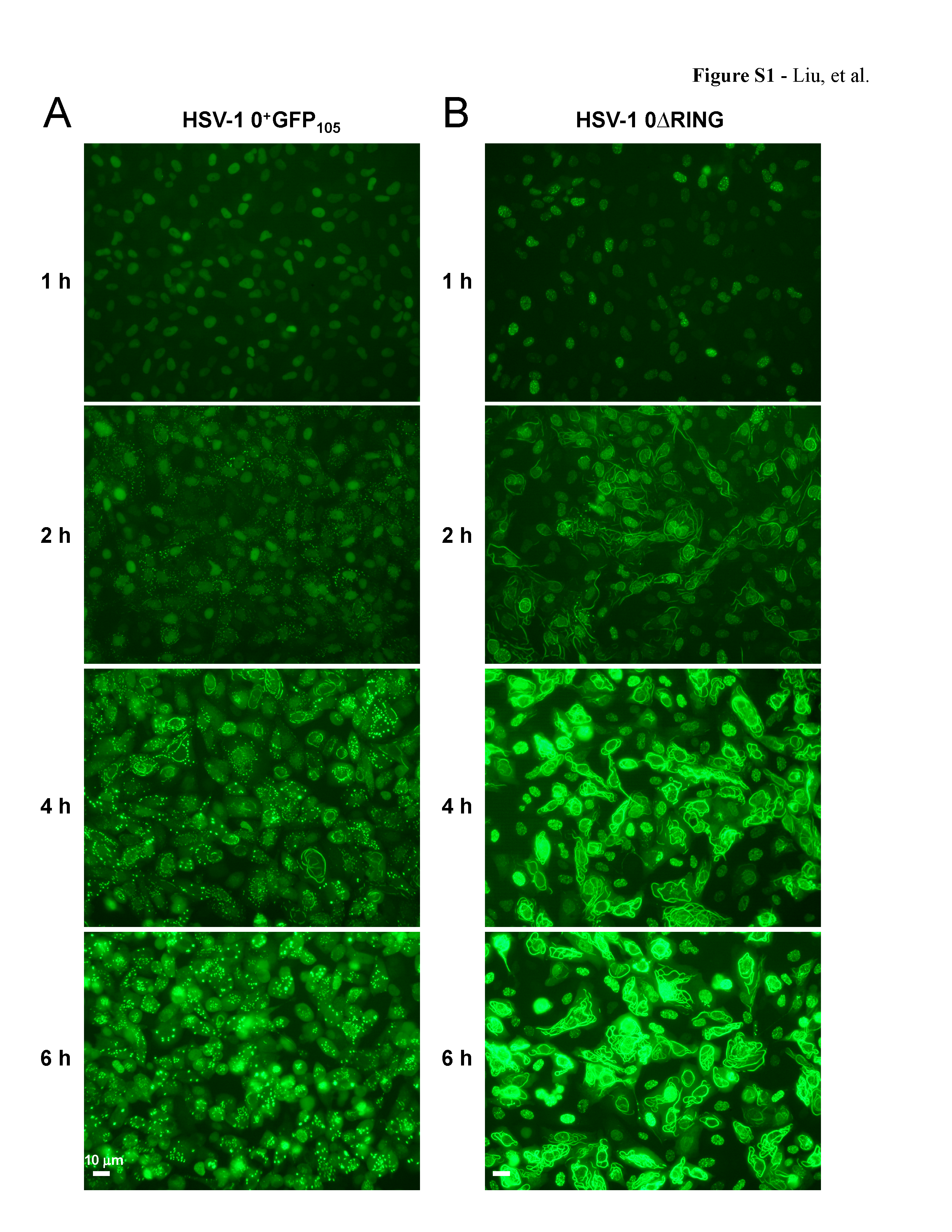

Supplement: Figure S1 — ICP0+GFP-105 disperses linear cytoplasmic structures in HSV-1 0+GFP105-infected cells. Vero cells were inoculated with 5 pfu per cell of (A) HSV-1 0+GFP105 or (B) HSV-1 0ΔRING in the presence of 200 µM cycloheximide from −0.5 to 10 hours p.i., and were released into medium containing no drugs. Photographs of (A) ICP0+GFP-105 or (B) ICP0ΔRING between 1 and 6 hours after release from the cycloheximide block. The scale bar denotes a distance of 10 µm. (6.55 MB TIF) [file pone.0010975.s001.tif]

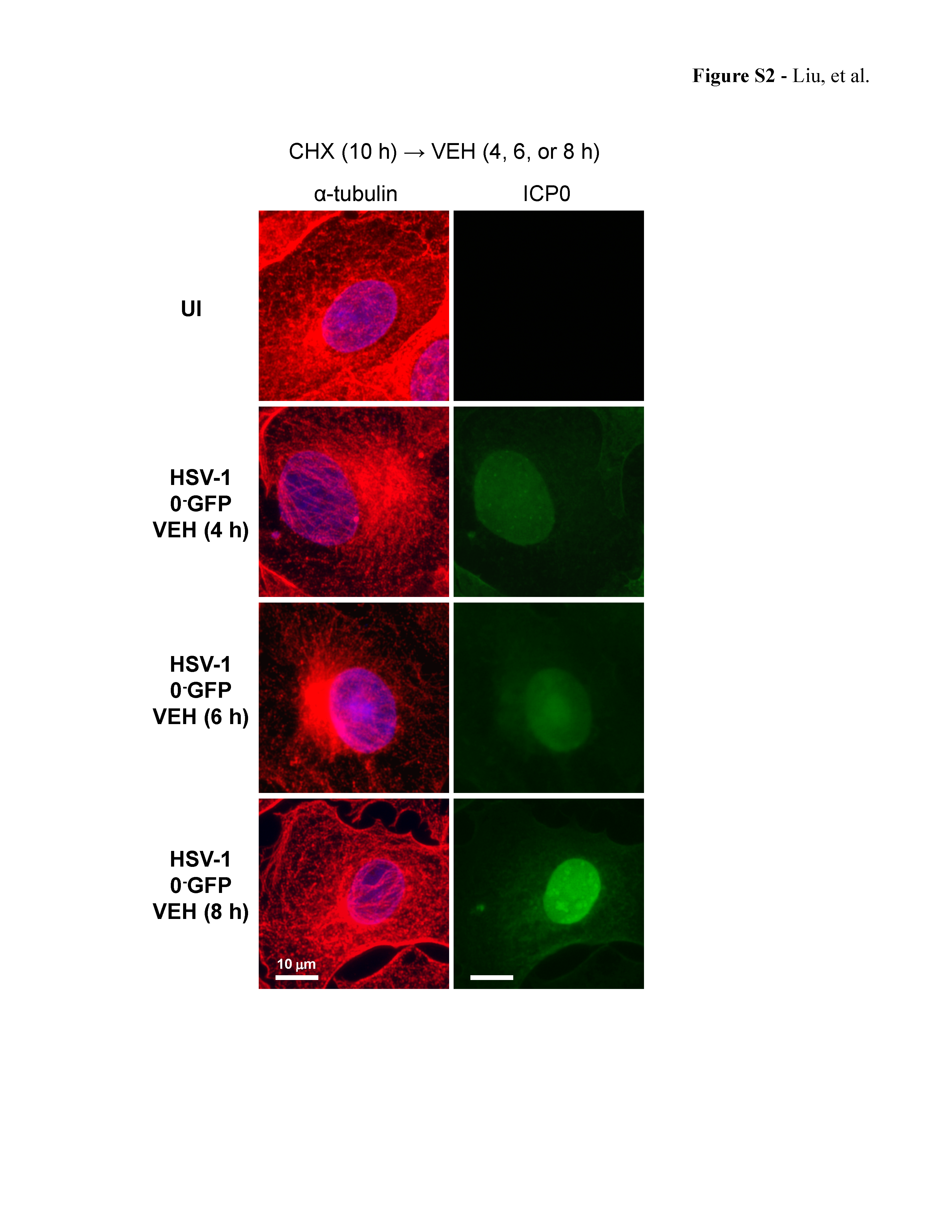

Supplement: Figure S2 — Microtubule networks remain intact in cells infected with an ICP0− null virus, HSV-1 0−GFP. Vero cells were uninfected or were inoculated with 5 pfu per cell of inoculated with HSV-1 0−GFP in the presence of 200 µM cycloheximide from −0.5 to 10 hours p.i., and were released into medium containing no drugs. At 4, 6, or 8 hours post-release, cells were fixed and stained with rabbit antibody against α-tubulin and the truncated ICP0−GFP peptide was directly visualized. The scale bar denotes a distance of 10 µm. (5.81 MB TIF) [file pone.0010975.s002.tif]

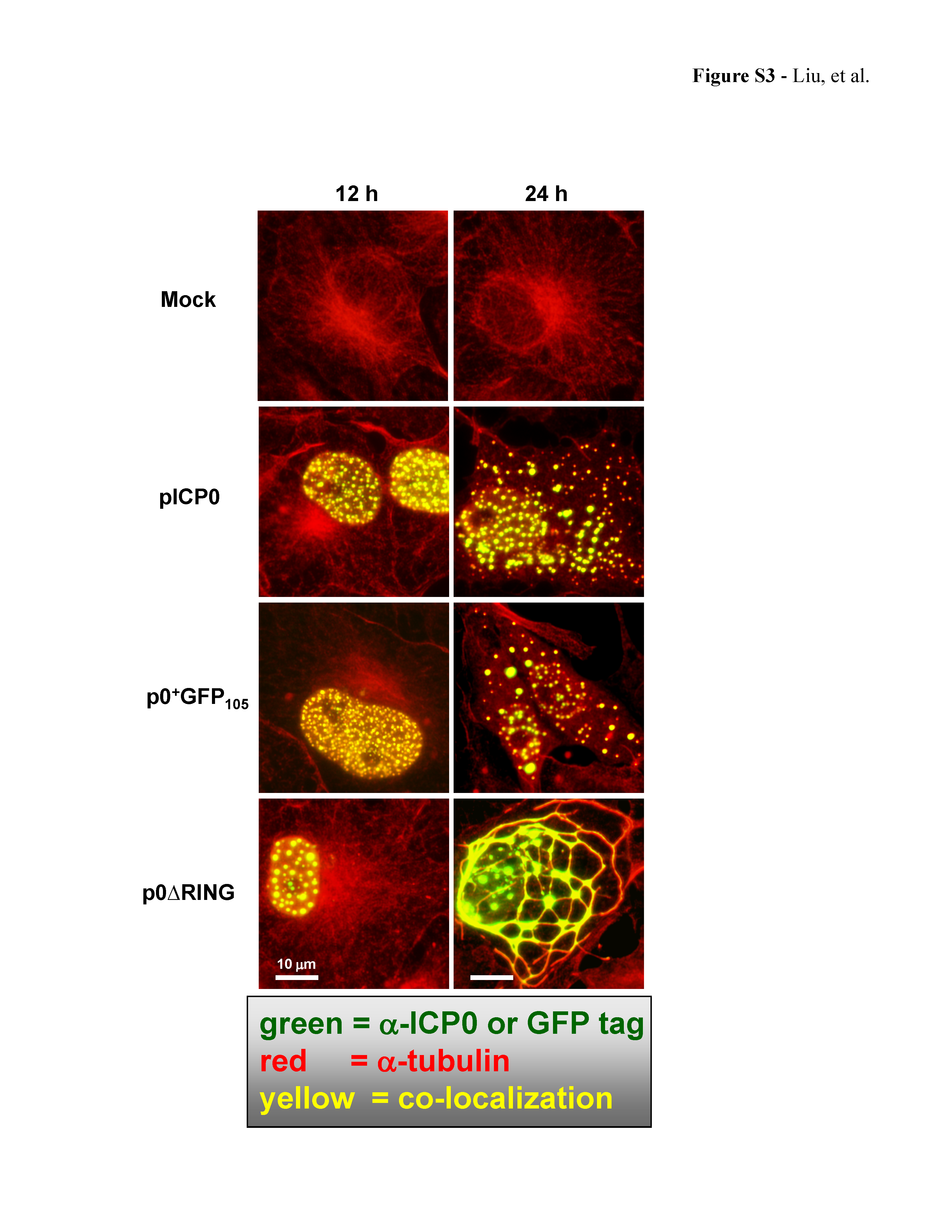

Supplement: Figure S3 — Co-localization of ICP0 and α-tubulin in cells transfected with ICP0-expressing plasmids. Merged images of the α-tubulin and ICP0 staining shown in Figure 9 in cells that were mock transfected, or which were transfected with pICP0, p0+GFP105, or p0ΔRING. The scale bar denotes a distance of 10 µm. (7.18 MB TIF) [file pone.0010975.s003.tif]

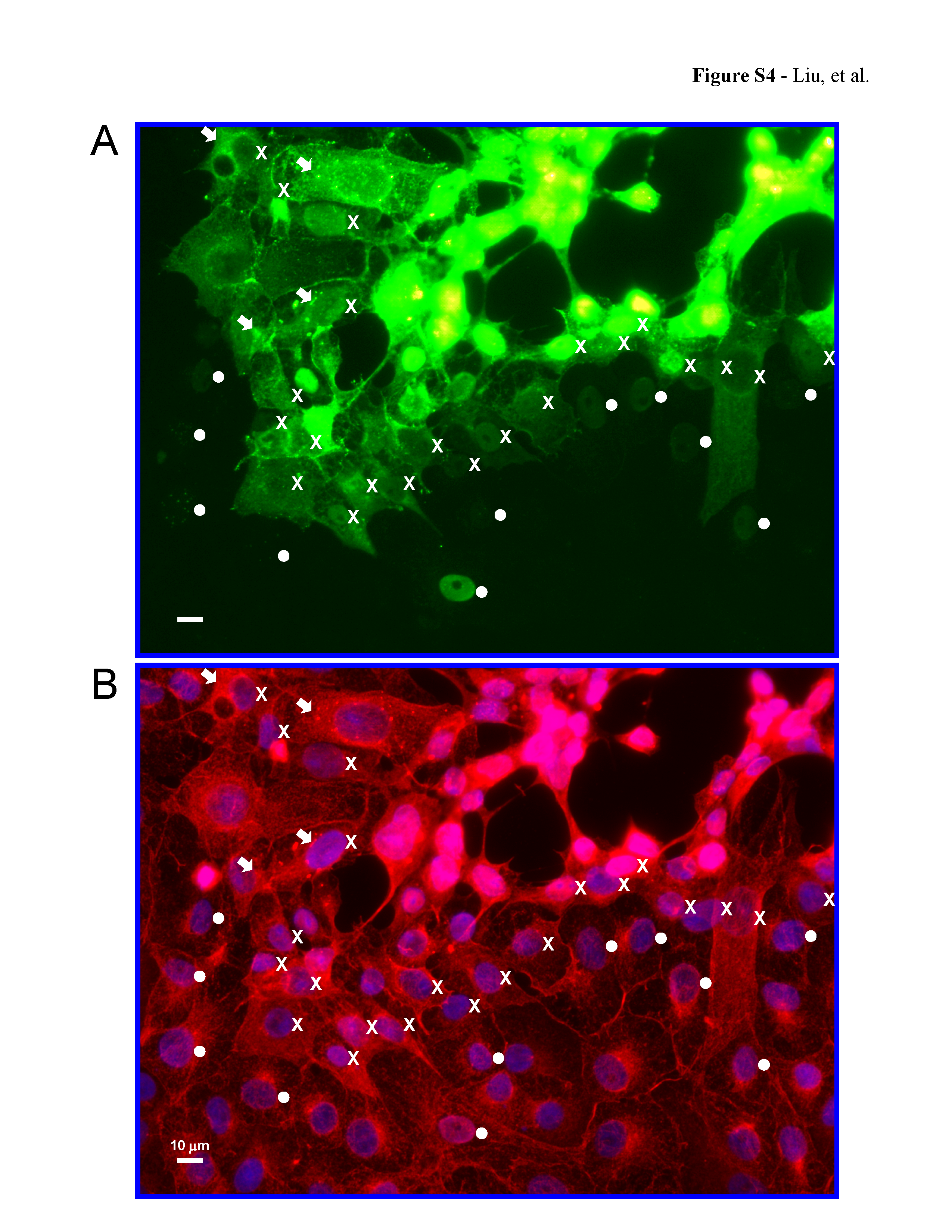

Supplement: Figure S4 — Enlarged image of one portion of an HSV-1 plaque in which ICP0 translocation, α-tubulin dispersal, and co-localization of ICP0 and α-tubulin are observed. Enlarged photographs of (A) ICP0-staining and (B) α-tubulin staining from Figure 10. (7.30 MB TIF) [file pone.0010975.s004.tif]
